# Identification of hsa-miR-144-3p as a novel immunotherapeutic target for glioblastoma based on disulfidptosis-related analysis

**DOI:** 10.1007/s12672-026-05048-3

**Published:** 2026-04-26

**Authors:** Mengda Li, Juntao Li, Ye Yuan, Zhixiao Li, Guanzheng Liu, Yongji Guo, Yuanhang Zhou, Zikuan Chai, Chao Wang, Xudong Fu, Xingyao Bu, Xiao-Tang Kong, Chunxiao Ma

**Affiliations:** 1 Department of Neurosurgery, Henan University People’s Hospital, Zhengzhou, 450000 China; 2 Department of Neurosurgery, Henan Provincial People’s Hospital, No. 7, Wei Wu Road, Jinsui District, Zhengzhou, 450000 Henan China; 3 Department of Neurosurgery, Union Hospital, Tongji Medical College, Huazhong University of Science and Technology, Wuhan, 430000 China; 4 Clinical Medicine College, Zhengzhou University, Zhengzhou, 450000 Henan China; 5 Cardiothoracic Surgery, Xinhua Hospital Affiliated to Shanghai Jiao Tong University School of Medicine, Shanghai, 200092 China; 6 Department of Neurosurgery, Zhengzhou University People’s Hospital, Zhengzhou, 450000 China; 7 Department of Neurology, UC Irvine Health, UCI Medical Center, Pavilion 1, 101 The City Drive South, Building 30, Orange, CA 92868 USA

**Keywords:** Glioblastoma, Disulfidptosis, TCGA, GEO, Hsa-miR-144-3p, Immune cell

## Abstract

**Background:**

There is no relevant research on the effect of disulfidptosis-related microRNA (miRNA) on the immune microenvironment in glioblastoma (GBM).

**Method:**

We integrated transcriptomic data from The Cancer Genome Atlas (TCGA), Gene Expression Omnibus (GEO) and Chinese Glioma Genome Atlas(CGGA) databases to comprehensively identify disulfidptosis-related miRNAs in GBM and evaluated their functional pathways and immunological relevance using bioinformatics approaches.

**Result:**

We identified hsa-miR-144-3p as a key disulfidptosis-related miRNA significantly associated with GBM. Functional analyses revealed that miR-144-3p was markedly enriched in immune-related pathways and strongly correlated with stromal score, immune score, and tumor purity. Moreover, its expression was significantly associated with immune checkpoint expression and immune cell infiltration patterns, suggesting a pivotal role in modulating the tumor immune microenvironment.

**Conclusions:**

This study reveals that the disulfide-related gene miR-144-3p, as a potential key miRNA may improve the prognosis by regulating immune cell infiltration and promoting the expression of immune checkpoints in GBM and serve as a target in GBM treatment. Our findings also provide new insights into the mechanism of disulfidptosis in GBM.

**Supplementary Information:**

The online version contains supplementary material available at 10.1007/s12672-026-05048-3.

## Background

GBM is the most aggressive and lethal primary brain malignancy in adults, and carries a dismal prognosis [1], even with standard first-line treatment consisting of maximal surgical resection followed by radiotherapy and temozolomide (TMZ) chemotherapy [2]. Recent evidence has elucidated the critical role of the immunosuppressive tumor microenvironment (TME) and immune evasion mechanisms in GBM progression [3]. Consequently, investigations into TME-regulatory genes to provide theoretical support for novel therapeutic strategies have become imperative.

Disulfidptosis, a newly discovered form of metabolic cell death, plays a fundamental role in cancer therapy. Elevated disulfidptosis activity has been demonstrated in low-grade gliomas(LGG), GBM, and prostate adenocarcinoma. Especially, neurological malignancies (including LGG and GBM) exhibit the highest disulfidptosis scores [4]. Disulfidptosis, a metabolically regulated cell death modality [5], occurs when SLC7A11 overexpression during glucose deprivation excessively depletes intracellular nicotinamide adenine dinucleotide phosphate(NADPH), thereby inducing disulfide stress and aberrant disulfide bond in actin cytoskeletal proteins, ultimately resulting in cytoskeletal collapse and cell death [6]. The concept was first introduced by Professor Gan Boyi in 2017 [7], and in the same year, Katoh Hironori et al. proposed a potential role of disulfidptosis in GBM [8]. Disulfidptosis has also been implicated in modulating redox signaling and immune responses, potentially contributing to improved cancer prognosis. Its interplay within the TME becomes pivotal for understanding cancer progression, metastasis, and therapeutic intervention [9]. However, its relevance to GBM—particularly regarding immune infiltration and cell–cell interactions—remains unclear.

MiRNAs are small non-coding RNAs that regulate gene expression at the post-transcriptional level. While the fundamental principles of miRNA biogenesis and function have been established, recent breakthroughs have uncovered critical new insights into their molecular regulation [10]. miRNAs have demonstrated diagnostic and therapeutic potential in various cancers [11], yet the field of miRNA-based therapy is still in its infancy. There is a growing need to understand the regulatory networks involving miRNAs in GBM for the identification and treatment of diseases [12].

**Fig. 1:**
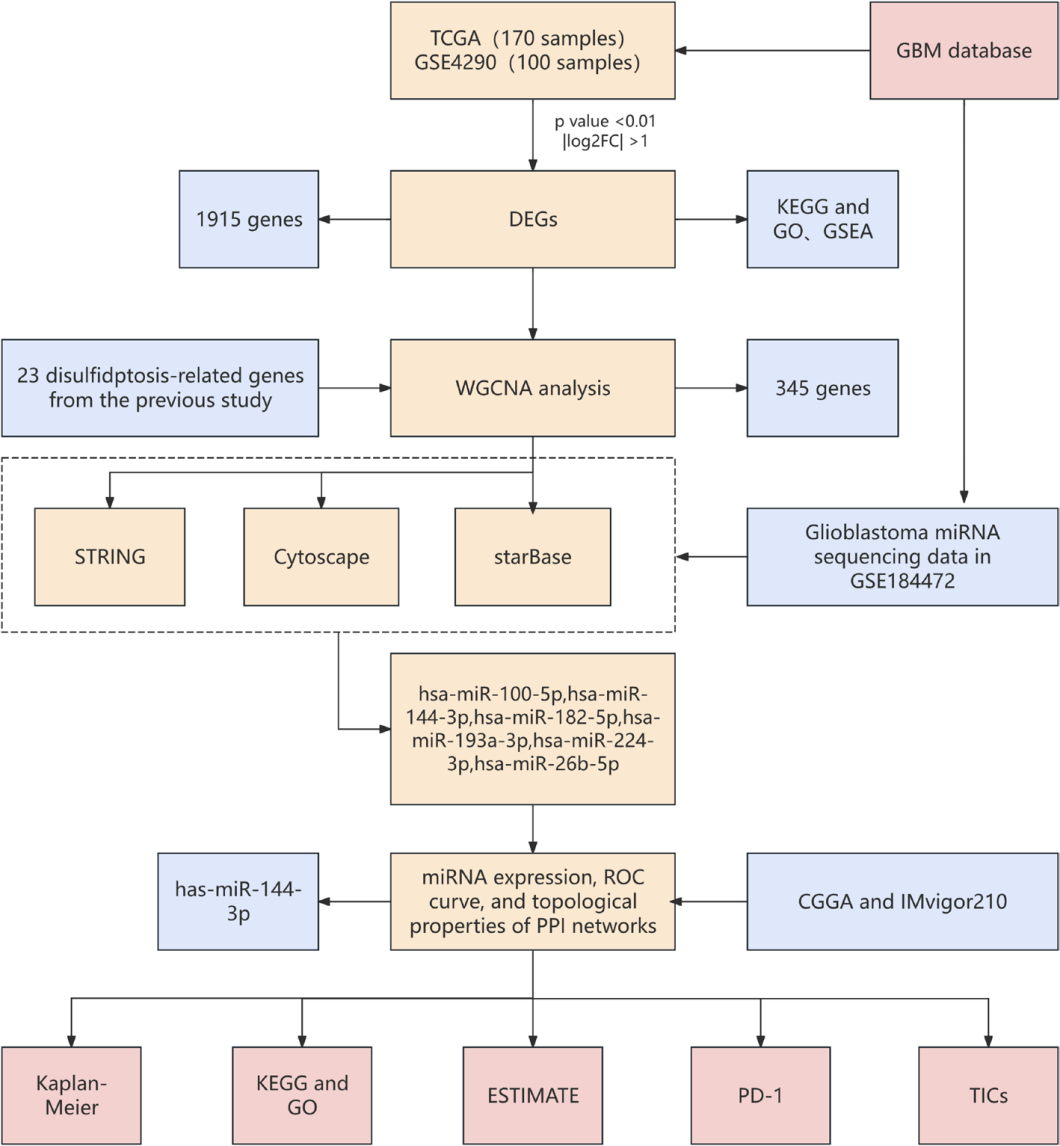
Analytical flow chart in this study

This study investigates the link between disulfidptosis and the GBM immune microenvironment using public datasets and aims to identify potential biomarkers and therapeutic miRNA targets. We systematically evaluated the roles of disulfidptosis-related genes in the GBM immune landscape.WGCNA was applied to identify genes associated with the disulfidptosis score, and hsa-miR-144-3p was further pinpointed as a target miRNA through database screening. Hsa-miR-144-3p demonstrated prognostic relevance and showed exploratory associations with immune-related features; however, its predictive value for immunotherapy response remains to be validated in GBM-specific cohorts. Mechanistically, hsa-miR-144-3p appears to enhance the prognosis of GBM patients by modulating immune cell infiltration and receptor expression in coordination with disulfidptosis. The analytical flowchart of this study is displayed in Fig. 1. Such findings highlight the therapeutic potential of targeting hsa-miR-144-3p in the treatment of GBM.

## Materials and methods

### Download and preprocessing of gene expression profiles

RNA-sequencing data, overall survival, and clinical details of GBM patients were downloaded from TCGA via the UCSC Xena browser (https://xena.ucsc.edu). Additionally, the GSE4290 [13] and GSE184472 [14] were retrieved from the GEO portal (https://portal.gdc.cancer.gov/). The TCGA dataset included 170 GBM tumor samples and 5 normal brain tissue samples. In GEO portal, GSE4290 comprised 77 GBM tumor samples and 23 normal brain samples, while GSE184472 consisted of 45 GBM tumor samples and 73 normal samples. Notably, TCGA and GSE4290 contained mRNA expression data, whereas GSE184472 provided miRNA expression data (Table 1).

**Table 1:** Dataset information

Accessions	Platforms	Samples (tumor vs. nontumor tissues)	References
TCGA	Illumina	170 vs. 5	Cancer Genome Atlas Research Network [15]
GSE4290	GPL570	77 vs. 23	Sun et al. [13]
GSE184472	GPL15520 GPL16791	45 vs. 73	Bustos et al. [14]

To reduce potential bias arising from cross-platform heterogeneity, differential expression analyses were performed independently within each dataset rather than by directly merging raw expression matrices from different platforms. For microarray datasets, probes corresponding to the same gene symbol were summarized by their average expression value. Expression matrices were log2-transformed when necessary and normalized within each dataset according to the preprocessing framework applicable to the original platform.

### Differential analysis

Differentially expressed genes (DEGs) were detected using the limma package (version 3.62.1) following log2 transformation. The thresholds for statistical significance were set at adjusted *p* < 0.01 and | log2FC | > 1. Volcano plots and principal component analysis (PCA) plots were produced via the ggplot2 package to visualize expression patterns and sample clustering. After quality assessment using PCA, robust differentially expressed genes were defined as the intersection of significant genes identified independently in the TCGA and GSE4290 cohorts. This strategy was used to minimize platform-specific effects and retain consistently dysregulated genes across cohorts. Because GSE184472 provided miRNA expression data generated from a different platform, differential miRNA analysis was likewise conducted independently and used only for downstream intersection with predicted miRNA candidates, rather than for direct cross-platform expression merging.

### Enrichment analysis

The clusterProfiler package (version 4.2.2) was employed to carry out pathway enrichment analyses of Gene Ontology (GO) [16] and Kyoto Encyclopedia of Genes and Genomes (KEGG) [17] on the identified DEGs. The KEGG database is a comprehensive resource that integrates information on genomes, biological pathways, diseases, drugs, and other relevant domains, whereas the GO database serves as a standardized framework for classifying and annotating gene functions [16, 17]. All enrichment analyses were corrected for multiple testing using the false discovery rate (FDR). These analyses facilitate the elucidation of the roles of these genes in biological processes, molecular functions, and cellular components, as well as their involvement in metabolic and signaling pathways.

### Disulfidptosis score calculation

A previously published scoring model was used to quantify the disulfidptosis level in GBM, defined as the difference between enrichment scores of genes promoting and inhibiting disulfidptosis [4]. A total of 23 genes associated with disulfidptosis were categorized into two groups. The first group is pro-disulfidptosis genes (1): SLC7A11, SLC3A2, RPN1, NCKAP1, CYFIP1, WAVE2, ABI2, HSPC300, RAC1, ATF4; and the second group is anti-disulfidptosis genes (2): NUBPL, NDUFA11, LRPPRC, OXSM, NDUFS1, GYS1, G6PD, PGD, TALDO1, TKT, PRC1, GLUT1, GLUT3 [6, 18, 19]. The single-sample Gene Set Enrichment Analysis (ssGSEA) algorithm, implemented in the “GSVA” package, was used to calculate enrichment scores for both gene sets [20]. ssGSEA extends the classical GSEA approach to individual samples, enabling the conversion of gene expression matrices into sample-specific enrichment profiles. This technique allows functional interpretation of cellular states based on the activity of biological processes and signaling pathways [21, 22].

### Construction of WGCNA

The WGCNA package was used to calculate gene–gene correlations and to construct a weighted co-expression network based on the differentially expressed genes. The soft-thresholding power (β) was selected according to the scale-free topology criterion. Specifically, candidate powers were evaluated based on both the scale-free topology fit index and mean connectivity, and the lowest power at which the scale-free topology fit index approached 0.90 was selected. A weighted adjacency matrix was then constructed from pairwise correlations and further transformed into a topological overlap matrix (TOM). Hierarchical clustering was performed based on TOM dissimilarity, and modules were identified using the dynamic tree cut algorithm, with the minimum module size set to 10 genes. Highly similar modules were merged using a cut height of 0.25. Module eigengenes were subsequently calculated, and Pearson correlation analysis was performed to assess the associations between module eigengenes and the disulfidptosis score.

### Construction of the disulfidptosis-related miRNA-mRNA network in GBM

The genes were uploaded to the STRING (v12.0) database to construct the protein–protein interaction (PPI) network (species: Homo sapiens; minimum required interaction score = 0.4; hide disconnected nodes) [23]. Regulatory relationships between miRNAs and mRNAs were downloaded from the starBase database and overlapped with DEGs from the GSE184472 dataset [24]. The resulting network was exported and imported into Cytoscape (v3.10.4). for further topological analysis using the NetworkAnalyzer plugin. Network topology parameters, including degree value and average shortest path length, were calculated for the target-gene networks corresponding to candidate miRNAs. Highly connected subnetworks were identified using the MCODE plugin with the following parameters: degree cutoff = 2, node score cutoff = 0.2, k-core = 2, and max depth = 100. Modules with network scores greater than 5.0 were considered significant and subjected to subsequent KEGG enrichment analysis.

### Receiver operating characteristic (ROC) analysis of key regulatory miRNAs

ROC curve analysis was employed to assess the diagnostic performance of key miRNAs. The ‘roc’ function from the pROC package was used, inputting actual labels and predicted scores. By plotting the ROC curve, sensitivity and specificity at various thresholds were visualized. The area under the curve (AUC) was calculated to quantify model performance, with values ranging from 0 to 1, where 1 suggests perfect classification and 0.5 suggests no classification capability.

### External validation and independent prognostic assessment

To minimize potential overfitting and enhance the robustness of the model, during the external validation process of the CGGA cohort, the same risk scoring formula and critical thresholds derived from the TCGA cohort were directly applied, and the validation cohort was ensured to remain completely independent. Meanwhile, KM survival analysis was used to evaluate the association between PC1 grouping and patients’ survival time. Multivariate Cox regression analysis was performed in both cohorts to adjust for identified clinical prognostic factors, thereby reducing confounding bias and confirming the independent prognostic value of this feature. These steps collectively reduce the risk of overfitting and improve the reliability of the model’s predictive performance in independent populations. Due to the absence of publicly available GBM cohorts with transcriptomic profiles and matched immunotherapy response data, we utilized the IMvigor210 anti–PD-L1 cohort as an exploratory cross-cancer validation dataset to explore whether the has-miR-144-3p–related signature captures immune-associated biological signals beyond GBM, rather than to provide disease-specific validation of immunotherapy response prediction. A signature score was calculated based on the expression of has-miR-144-3p–associated target genes. The relationships between the signature score and treatment response, overall survival, and clinical variables were assessed using ROC analysis, Kaplan–Meier survival analysis, and multivariate Cox regression models.

### Validation of candidate biomarkers and predicted genes

To evaluate the prognostic potential of predicted genes, optimal cutoff points were determined based on overall survival and event status from TCGA samples for survival analysis grouping. Kaplan-Meier analysis and log-rank tests were conducted on 169 TCGA samples, incorporating clinical information and immunohistochemical results from the Human Protein Atlas(HPA) [25].

### Investigation of the tumor immune microenvironment

To further probe into the correlation between key miRNA sets and the immune microenvironment, the first principal component of key mRNA sets was calculated using PCA. Samples were divided into high and low PC1 groups by the median value. The ssGSEA enrichment analysis from the GSVA package was utilized to assess tumor-infiltrating immune cells (TICs) in GBM cases within TCGA. Box plots were generated to reveal differences in 28 TICs from the TISIDB database across groups. Meanwhile, we used CIBERSORT to estimate the proportion of immune cell infiltration in the samples, and further validated the results by displaying the differential distribution of different immune cells in different groups through box plots. Additionally, stromal, immune, tumor purity scores, and expression levels of immune checkpoint proteins PD-L1 and PD-1 (encoded by CD274 and PDCD1, respectively), were examined. Finally, we calculated the correlation between PC1 and several key immune indicators.

## Result

### Detection of DEGs in GBM

**Fig. 2:**
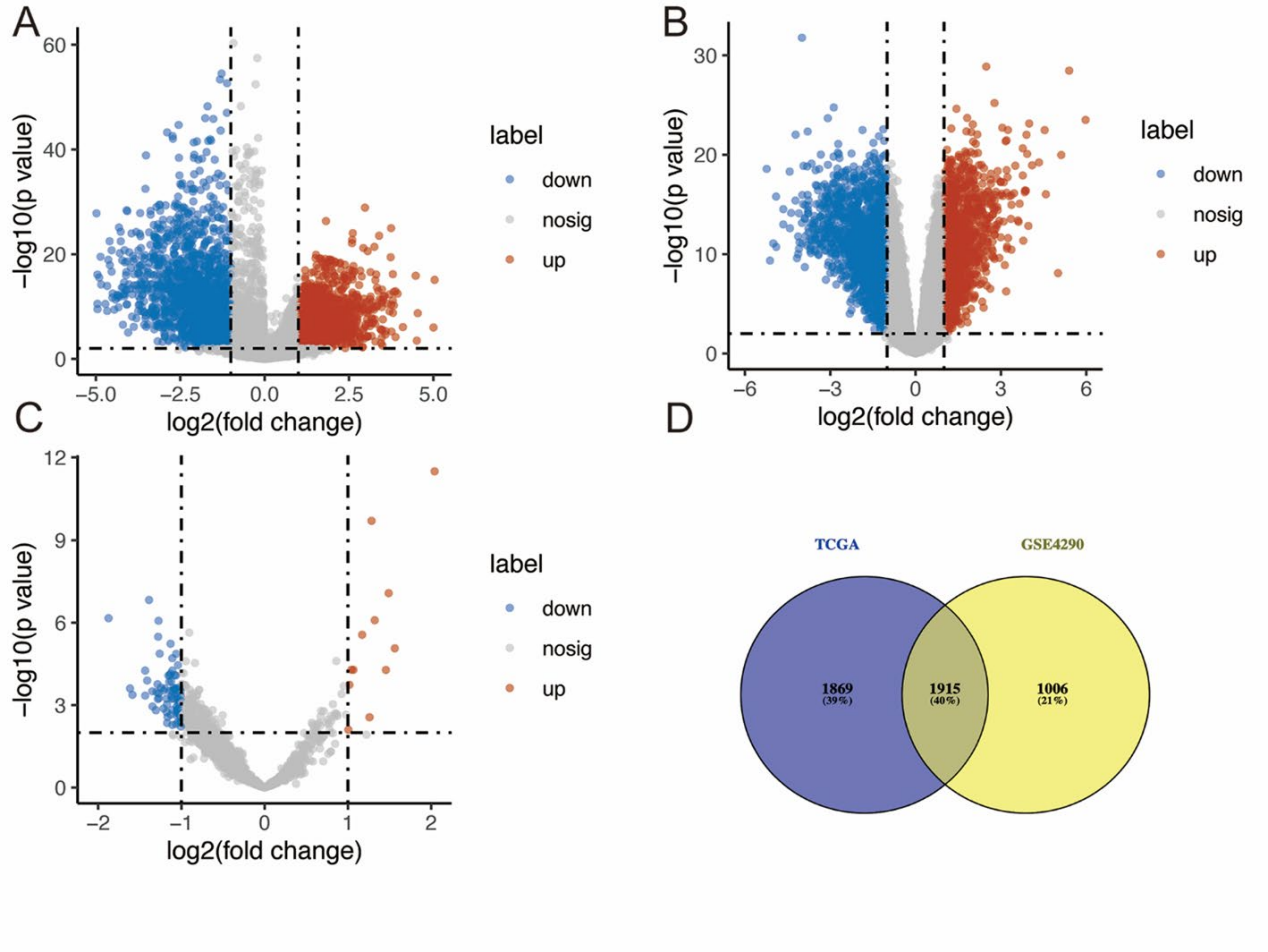
Detection of DEGs in GBM datasets. **A**–**C** Number of upregulated and downregulated genes in GSE4290, TCGA, and GSE184472; red dots represent upregulation in tumors compared to normal tissues, blue dots indicate downregulation, and gray dots mean no significant difference. **D** Venn diagram of DEGs between GSE4290 and TCGA

In the TCGA dataset, 1,954 upregulated and 1,830 downregulated mRNAs were identified (Table S1) (Fig. 2A). In the GEO dataset GSE4290, 1,410 upregulated and 1,511 downregulated mRNAs were found (Table S2) (Fig. 2B, Figure S1). In the GEO dataset GSE184472, 12 upregulated and 80 downregulated miRNAs were detected (Table S3) (Fig. 2C). To identify high-confidence DEGs, the intersections of DEGs from TCGA and GSE4290 datasets were taken, resulting in 1,915 DEGs (Table S4) (Fig. 2D).

### Functional enrichment analysis of DEGs in GBM

**Fig. 3:**
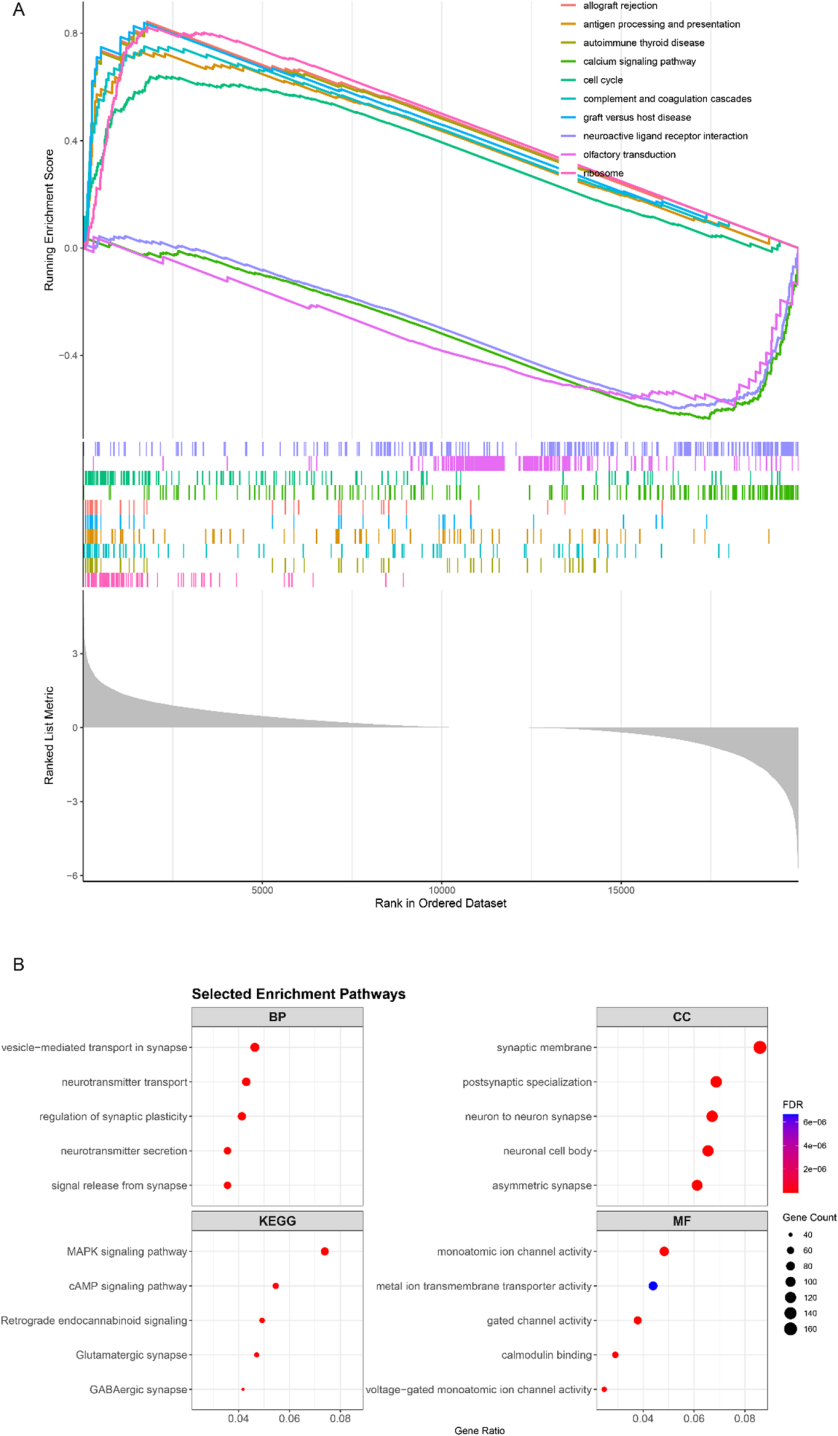
Enrichment analysis of 1915 DEGs in GBM. **A** GSEA analysis of DEGs; statistical significance assessed using one-sided t-test. **B** GO and KEGG analysis of DEGs. GSEA: Gene Set Enrichment Analysis

GSEA was performed on 1915 DEGs to clarify biological signaling pathways (Fig. 3A). Among the top 10 significantly enriched KEGG pathways were allograft rejection, antigen processing and presentation, autoimmune thyroid disease, calcium signaling pathway, cell cycle, complement and coagulation cascades, graft-versus-host disease, neuroactive ligand-receptor interaction, olfactory transduction, and ribosome, all of which were significantly activated in GBM (adjusted P-value < 0.05). GO enrichment analysis revealed that DEGs were involved in the modulation of chemical synaptic transmission, synaptic membrane, and monatomic ion channel activity (Fig. 3B). KEGG analysis revealed enrichment in MAPK-signaling pathway and cAMP signaling pathway (Fig. 3B).

### Construction of disulfidptosis score-related module genes

**Fig. 4:**
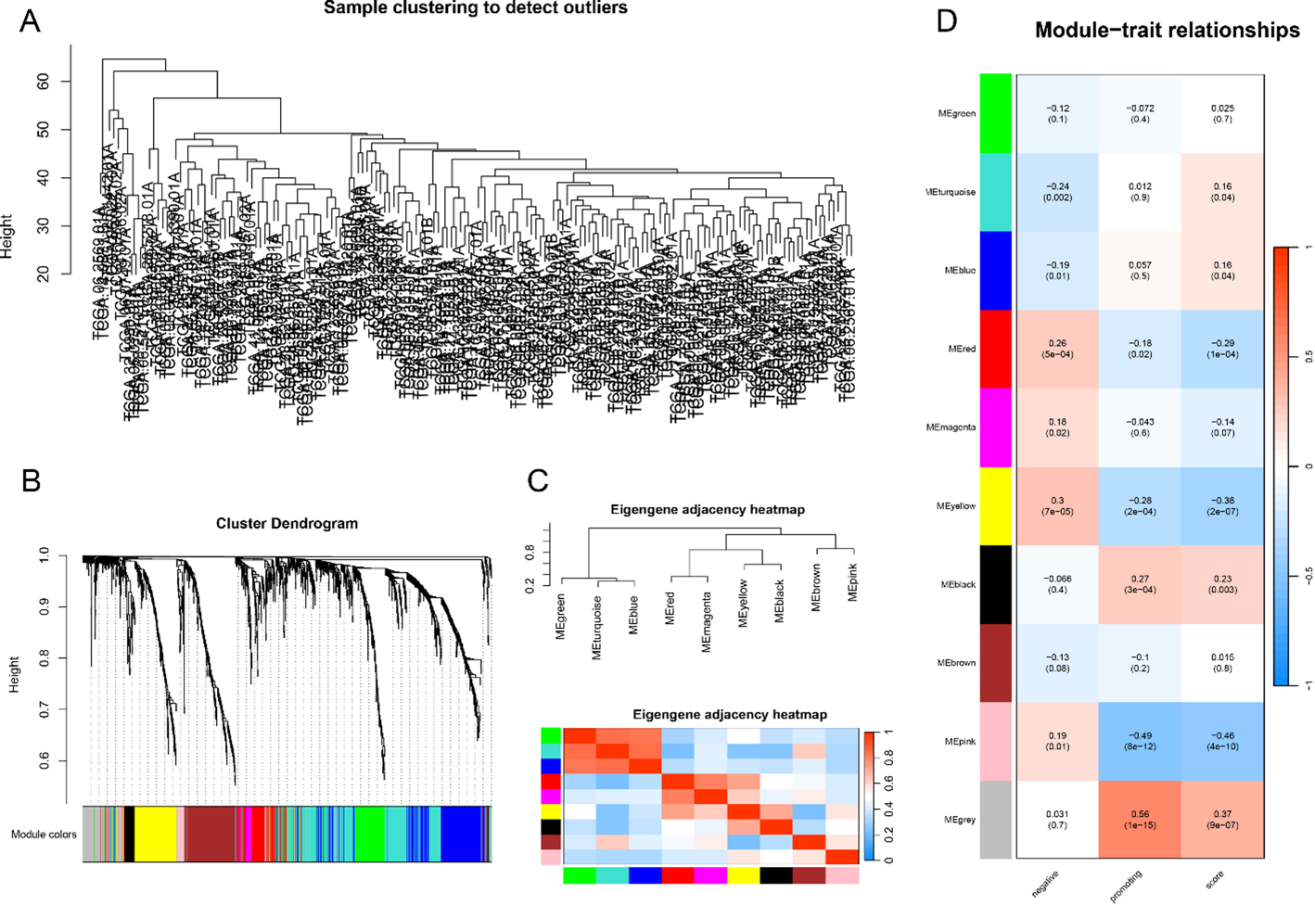
Construction of WGCNA in GBM datasets. **A** Clustering dendrogram of 169 samples. **B** Clustering dendrogram of genes. **C** Gene dendrogram generated through average linkage hierarchical clustering. **D** Module-trait relationship

Sample grouping did not reveal any outliers (Fig. 4A). To determine an appropriate soft-thresholding power for network construction, candidate powers were evaluated using the scale-free topology fit index (R²) and mean connectivity. A soft threshold of 6 was selected because it was the lowest power at which the scale-free topology model fit index approached 0.9 (Figure S2A), while the mean connectivity was reduced to near zero (Figure S2B), indicating that the resulting network adequately approximated a scale-free topology. Hierarchical clustering analysis based on co-expression relationships (Fig. 4B) identified nine gene modules (Fig. 4C), labeled with different colors (green, turquoise, blue, red, magenta, yellow, black, brown, pink, grey) (Fig. 4D). Module eigengenes were used to construct a dendrogram, showing high heterogeneity among subgroups. Modules on the same branch and in close proximity were merged with a threshold of 0.25 to assess interactions. Following the acquisition of WGCNA network data, an evaluation of module-trait relationship was conducted. Specifically, the associations between each module and genes promoting disulfide formation, genes inhibiting disulfide formation, as well as the disulfidptosis score were analyzed. The results showed that yellow, pink, and grey modules had significant correlations with the disulfidptosis score. The grey module typically represents genes not classified into other modules, possibly due to inconsistent expression patterns or data noise, yet it correlated with the disulfidptosis score in this study. Therefore, 345 genes from these three modules were selected for in-depth analysis.

### Identification of hub genes and candidate miRNAs

**Fig. 5:**
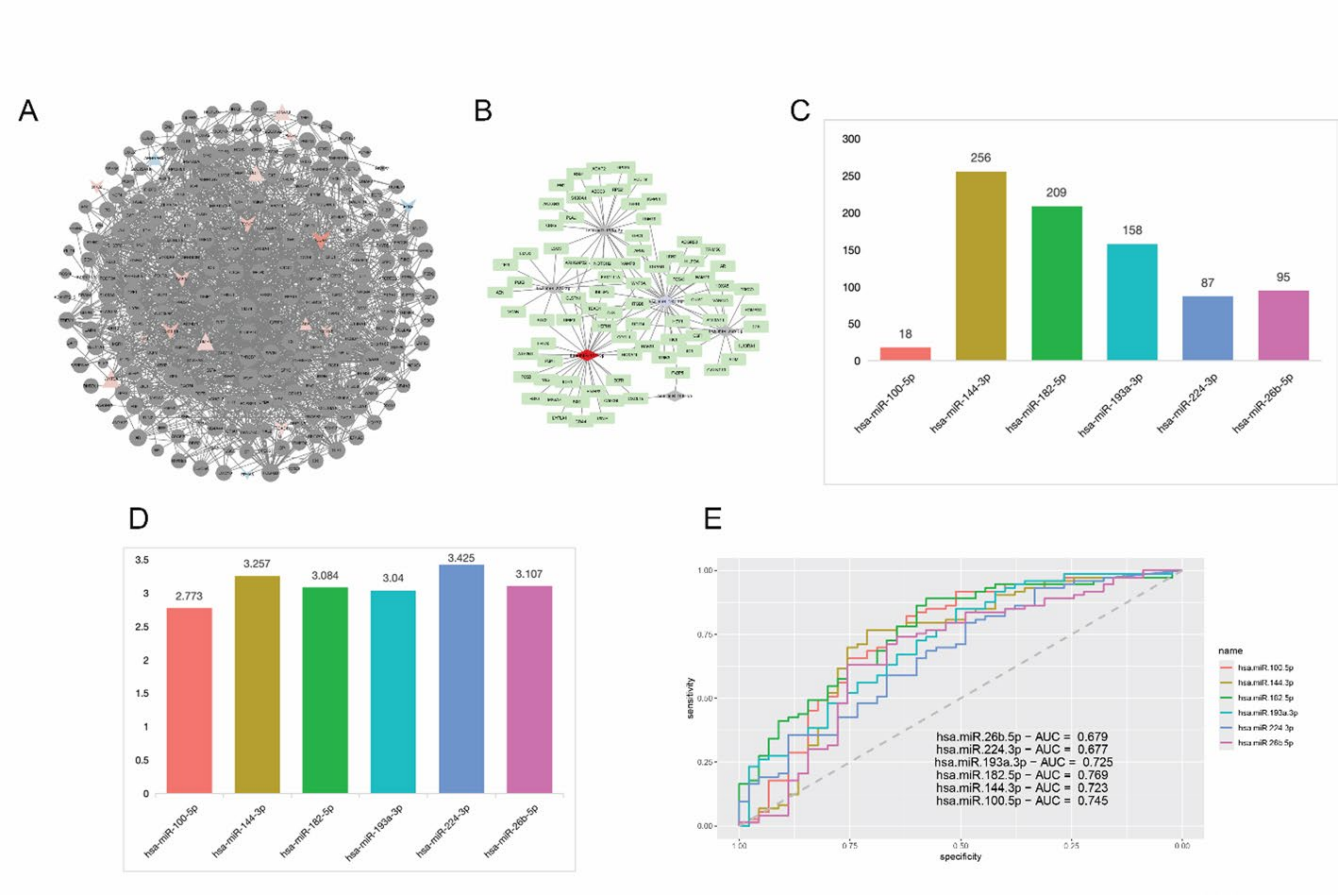
PPI network analysis and identification of key miRNAs. **A** PPI network of genes in the key module: the redder the circle, the higher the gene expression level; the larger the node, the shorter the average path length. The symbol “∨” represents genes negatively correlated with prognosis, and the symbol “△” represents genes positively correlated with prognosis; **B** PPI networks associated with the six candidate miRNAs; **C** Degree centrality of the six candidate miRNAs; **D** Degree centrality of the six candidate miRNAs; **E** ROC curves for the six miRNAs

The STRING database was utilized to construct the PPI network by inputting a total of 345 genes. The transcriptional expression levels of the corresponding mRNAs were mapped onto node color intensity, while the average shortest path was represented by node size (Fig. 5A, Table S5). Furthermore, MCODE application results were indicative of three significant modules. All network scoring of the three modules were more than 5.0. Module 1, module 2 and module 4 were significant modules in the PPI network. A total of 19 nodes and 99 edges were included in Module 1 (Figure S3A), and a total of 23 nodes and 71 edges were included in Module 2 (Figure S3B). In addition, there were 14 nodes and 35 edges in Module 4 (Figure S3C). Network properties were also analyzed. Importantly, functional clustering of its target gene network revealed a dominant enrichment in innate immune–related processes, particularly complement activation, phagocytosis, and neutrophil-mediated immune responses (Figure S4A–C). Based on mRNA–miRNA interactions obtained from the starBase database, miRNAs targeting genes in the PPI network were identified (Fig. 5B, Table S5). These candidate miRNAs were then intersected with differentially expressed miRNAs from the GSE184472 dataset, resulting in six overlapping miRNAs: hsa-miR-100-5p, hsa-miR-144-3p, hsa-miR-182-5p, hsa-miR-193-3p, hsa-miR-224-3p, and hsa-miR-26b-5p. The ROC results, including AUC, sensitivity, and specificity values for each miRNA, are summarized in Table S6. Among the six candidate miRNAs, hsa-miR-144-3p exhibited the highest diagnostic performance, together with favorable network topology characteristics(Table S7). Rather than representing broad immune activation, these findings suggest that miR-144-3p may serve as a regulatory hub coordinating key innate immune components within the GBM microenvironment. Based on these integrated criteria, hsa-miR-144-3p was identified as the core miRNA of interest (Fig. 5C–E).

To identify the key regulatory miRNA, multiple factors were integrated, including miRNA expression levels (adjusted *p* < 0.01 and | log2FC | > 1), topological metrics of their target mRNAs in the PPI network (degree value = 256, average shortest path length = 3.257), and diagnostic performance assessed by ROC analysis (AUC = 0.732, sensitivity = 0.767, specificity = 0.711). Among the six candidate miRNAs, hsa-miR-144-3p exhibited the highest overall diagnostic performance together with favorable network-topology characteristics, and was therefore selected as the core miRNA for subsequent analyses.

### Analysis of hsa-miR-144-3p and its target mRNAs

**Fig. 6:**
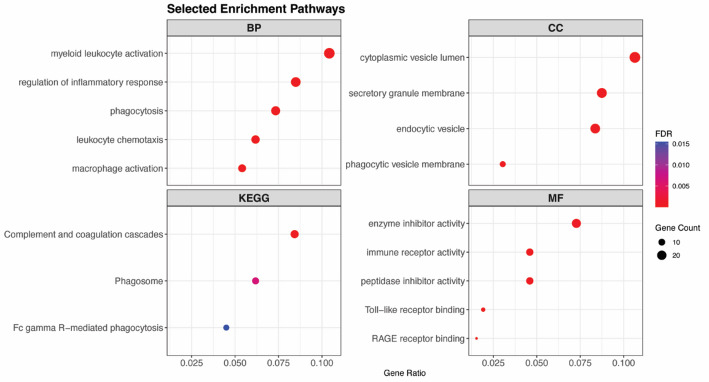
KEGG and GO enrichment analysis of the 30 target mRNAs

Functional enrichment analysis was performed on 30 predicted target mRNAs. Notably, these enrichments were primarily concentrated in complement and coagulation cascades, myeloid leukocyte activation, immunoglobulin binding, IgG binding, Toll-like receptor binding. These pathways are central regulators of immune cell activation and innate immune surveillance, supporting a potential role for has-miR-144-3p in modulating immune cell behavior within the GBM microenvironment (Fig. 6).

Furthermore, KEGG metabolic pathway analysis revealed enrichment in central carbon metabolism, amino acid biosynthesis, and glucose metabolism pathways (Figure S6A). Importantly, correlation analysis demonstrated that several has-miR-144-3p targets were strongly associated with disulfidptosis-related metabolic genes, including SLC7A11 and pentose phosphate pathway enzymes (Figure S6B). Given that metabolic reprogramming critically shapes immune cell activation and tumor immune escape, these findings suggest that has-miR-144-3p may influence immune microenvironment remodeling through coordinated regulation of metabolic–immune crosstalk.

### Survival analysis and immunohistochemistry

**Fig. 7:**
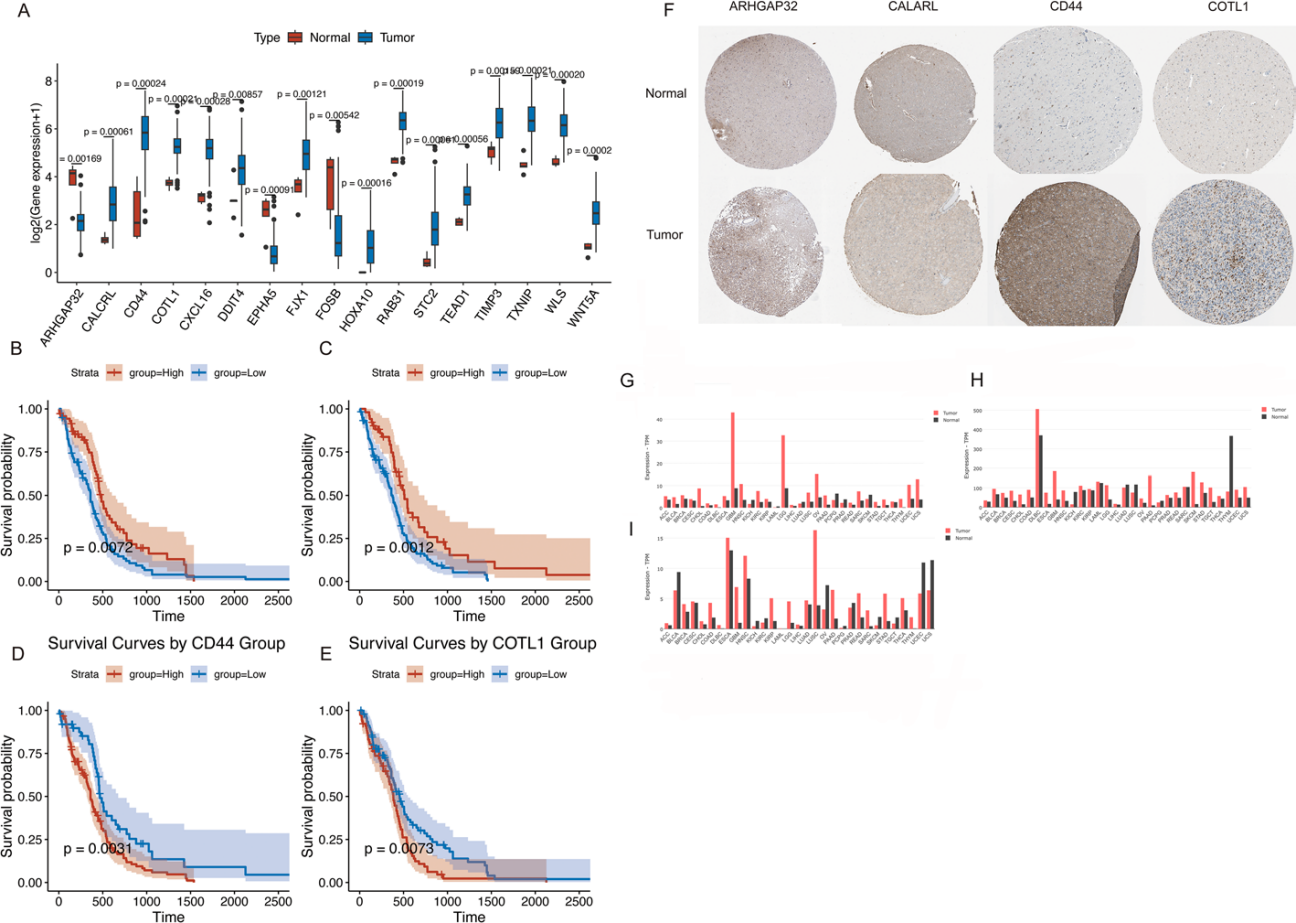
Expression, prognostic correlation, immunohistochemistry, and TPM analysis of related mRNAs. **A** Expression of the 30 target mRNAs in normal and tumor tissues; **B**–**E** Survival curves for RHGAP32, CALCRL, CD44, and COTL1; F: Immunohistochemical staining of RHGAP32, CALCRL, CD44, and COTL1; **G**–**I** Expression levels of FJX1, COTL1, and WNT5A in GEPIA

Survival analysis using TCGA data demonstrated that most genes targeted by hsa-miR-144-3p were significantly associated with patient prognosis. In total, 17 genes showed prognostic relevance (*p* < 0.05), of which 7 genes (ARHGAP32, CALCRL, DDIT4, HOXA10, TIMP3, WLS, WNT5A) were positively associated with prognosis, while 10 genes (CD44, COTL1, CXCL16, EPHA5, FJX1, FOSB, RAB31, STC2, TEAD1, TXNIP) were negatively associated with prognosis (Fig. 7A); additional results are provided in Figure S5. Notably, these 17 prognostic mRNAs also exhibited significant differential expression between tumor and normal tissues. The survival curves for genes with strong prognostic value (*p* < 0.01) are shown in Figs. 7B–E. The corresponding hazard ratios (HRs) with 95% confidence intervals (CIs) and p-values for each Kaplan–Meier analysis have been provided in Table 7. Immunohistochemical analysis using the HPA revealed that the protein expression patterns of ARHGAP32, CALCRL, and CD44 were consistent with their mRNA expression, while COTL1 showed opposite trends (Fig. 7F). Moreover, GEPIA analysis indicated that FJX1, COTL1, and WNT5A exhibited significantly elevated TPM values in tumor tissues (Fig. 7G–I).

### External validation of the PC1-based prognostic model in the CGGA cohort

To evaluate the robustness and generalizability of the PC1-based risk model, external validation was performed using the independent CGGA cohort. The same risk scoring formula and cutoff thresholds derived from the TCGA cohort were directly applied without modification to ensure strict independence between training and validation datasets.

**Fig. 8:**
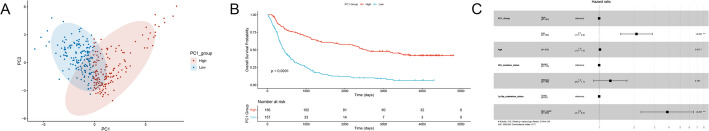
External validation in the CGGA cohort. **A** PCA plot showing clear separation between high and low PC1 groups; **B** Kaplan–Meier survival curves demonstrating significantly poorer overall survival in the high PC1 group; **C** multivariate Cox regression analysis confirming PC1 group as an independent prognostic factor

PCA analysis demonstrated clear separation between the high and low PC1 groups in the CGGA cohort, indicating stable stratification performance across independent populations (Fig. 8A). Kaplan–Meier survival analysis further revealed that patients in the high PC1 group exhibited significantly worse overall survival compared to those in the low PC1 group (*p* < 0.0001), consistent with the findings observed in the TCGA cohort (Fig. 8B).

To determine whether the PC1-based grouping served as an independent prognostic factor, multivariate Cox regression analysis was conducted after adjusting for established clinical variables, including age, IDH mutation status, and 1p/19q codeletion status (Fig. 8C). The results demonstrated that PC1 grouping remained an independent predictor of overall survival (HR = 2.1, *p* < 0.001).

In the IMvigor210 cohort, the has-miR-144-3p signature score was significantly higher in non-responders than in responders and demonstrated limited but statistically detectable predictive signal (AUC = 0.609), indicating weak discriminatory performance that does not meet thresholds for clinical utility (Figure S7A-B). Although no significant difference in overall survival was observed between high- and low-score groups (Figure S7C), multivariate Cox analysis confirmed the signature score as an independent prognostic factor after adjustment for clinical variables (Figure S7D). Nevertheless, the absence of survival stratification further underscores the exploratory nature of this cross-cancer analysis.

### ESTIMATE, and expression of immune checkpoints, and TICs

**Fig. 9:**
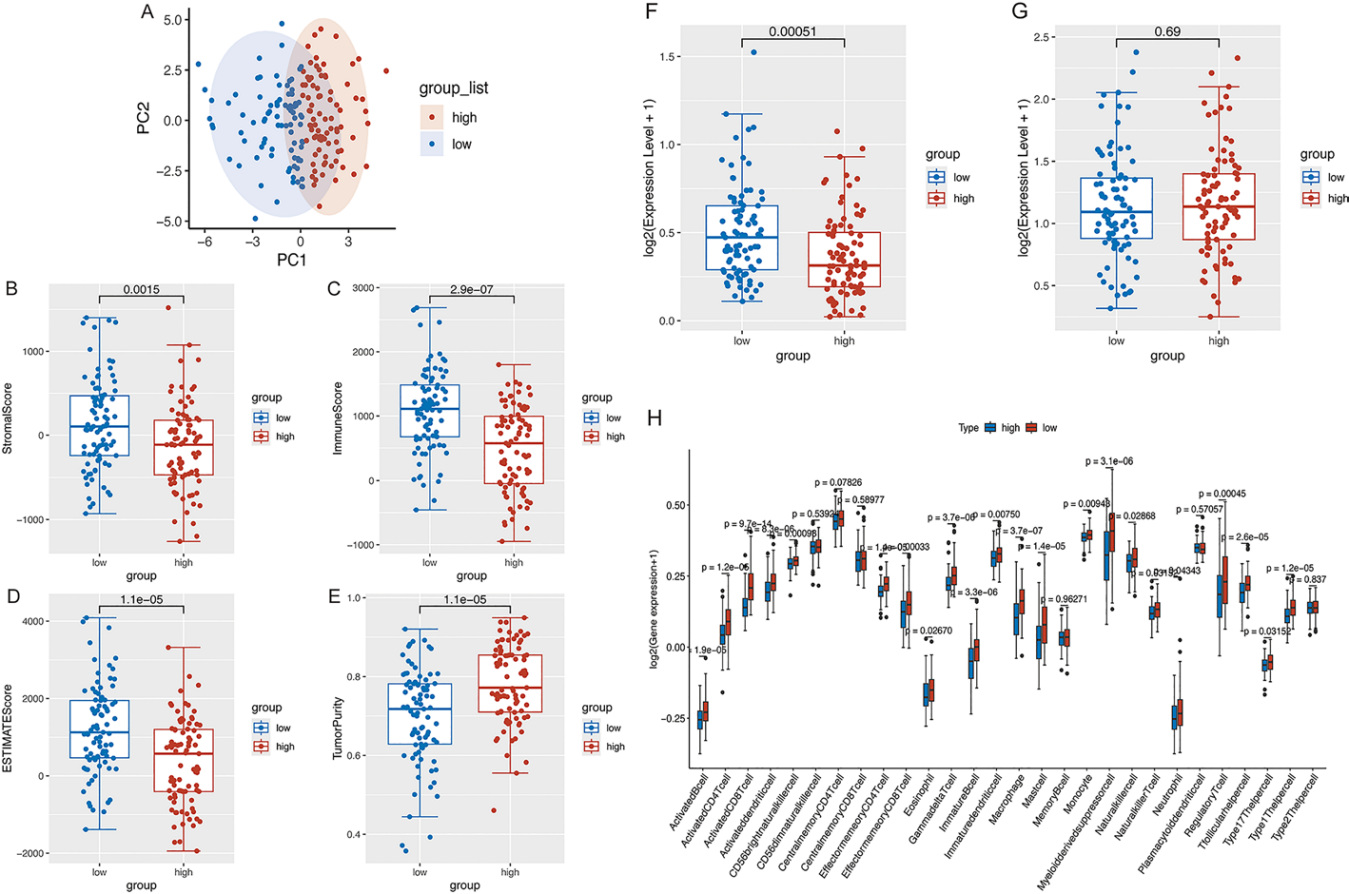
Tumor immune microenvironment analysis. **A** PCA-based stratification of tumor samples based on the 30 mRNAs; **B**–**E** ESTIMATE analysis; **F** PD1 (PDCD1) expression boxplot; **G** PDL1 (CD274) expression boxplot; **H** statistical significance was assessed using the Benjamini–Hochberg false discovery rate (FDR) method, and 22 of the 28 immune cell types demonstrated significant differences between groups (adjusted *p* < 0.05)

PCA was applied to the expression profiles of the target mRNAs of hsa-miR-144-3p, and samples were stratified into high and low PC1 score groups (Fig. 9A). To specifically evaluate the immune regulatory role of miR-144-3p–associated genes, we analyzed immune cell infiltration and immune checkpoint expression between groups. ESTIMATE analysis revealed that the low PC1 group exhibited significantly higher immune and stromal scores, together with reduced tumor purity, indicating a more immune-enriched microenvironment (Fig. 9B–E). Notably, PD-1 expression was significantly upregulated in the low PC1 group, while PD-L1 showed no significant difference (Fig. 9F–G). Additionally, immune checkpoints synergistic with PD-1, including CTLA4, HAVCR2, and VSIR, were significantly elevated in the PC1-low group (Figure S8A). These findings indicate that has-miR-144-3p–associated gene expression patterns are closely linked to enhanced immune checkpoint activity, suggesting potential implications for immunotherapeutic responsiveness in GBM. GSVA and CIBERSORT analyses suggested significant differences in inferred immune cell infiltration patterns between groups (Fig. 9H, Figure S8B-C). Specifically, the PC1-low group exhibited increased infiltration of activated CD8 + T cells, macrophages, monocytes, and γδ T cells, whereas the PC1-high group showed globally reduced immune infiltration (Table S8). PC1 was significantly negatively correlated with the immune score (*r* = -0.51, *P* < 0.05, Figure S9A) and also negatively correlated with the infiltration levels of various immune cells. Among them, the correlations with Activated CD8⁺ T cells (*r* = -0.69, *P* < 0.001, Figure S9B) and Macrophages (*r* = -0.52, *P* < 0.001, Figure S9C) were the most significant. These findings suggest that has-miR-144-3p–associated target genes are closely related to transcriptional features of the GBM immune microenvironment, particularly those linked to reduced inferred immune infiltration.

## Discussion

Disulfidptosis is triggered by NADPH depletion under glucose-starved conditions, leading to abnormal intracellular disulfide bond accumulation and cell death [6]. Activated immune cells increase glucose uptake, reduce oxidative phosphorylation and enhance ATP production to meet energy and biosynthetic demands for proliferation and pro-inflammatory responses [26]. GBM, characterized by an immunosuppressive tumor microenvironment [27] exhibits a high disulfidptosis score [4]. Thus, immune cells may collaborate with disulfidptosis by enhancing glucose consumption, thereby potentially improving GBM prognosis. Recent evidence further supports a mechanistic connection between disulfidptosis and immune evasion. Chen et al. demonstrated that redox-driven cytoskeletal collapse—a hallmark of disulfidptosis—can reshape the tumor immune microenvironment by impairing antigen presentation, altering T-cell activation, and promoting an immunosuppressive phenotype. Their study further revealed that SPAG4, a disulfidptosis-associated gene, enhances CD47 expression and suppresses macrophage phagocytosis, thereby facilitating immune escape [28]. These findings provide compelling evidence that disulfidptosis may contribute to immune evasion through redox imbalance and metabolic reprogramming. This study leveraged TCGA and GEO datasets to explore the regulatory roles of disulfidptosis-related miRNAs on the local immune microenvironment in GBM, thereby shedding light on their potential immunomodulatory functions. External validation using the CGGA and IMvigor210 datasets further confirmed the above conclusions. Based on integrative bioinformatics evidence, hsa-miR-144-3p was selected for further investigation.

Recent studies have increasingly combined immune-related transcriptomic features with advanced computational frameworks to improve tumor stratification and therapeutic prediction. For example, recent studies have used immune cell characteristics combined with machine learning or deep learning methods to predict the efficacy of immunotherapy [29], while other studies have adopted multimodal strategies that integrate genomic, transcriptomic, immune, and pathological image data to improve prognostic stratification [30], or used network toxicology and molecular docking to identify risk factors [31]. Compared with these approaches, the present study is distinguished by its focus on a disulfidptosis-related miRNA-centered framework in GBM. Rather than constructing a purely predictive multimodal classifier, our work integrates cross-cohort differential analysis, WGCNA, PPI topology, miRNA–mRNA regulatory inference, immune-microenvironment analysis, and external validation to identify hsa-miR-144-3p as a candidate molecule associated with both metabolic stress signaling and immune-related transcriptional states. This disease-focused and mechanism-oriented design provides a complementary contribution to recent biomarker-modeling studies.

GSEA of the differentially expressed genes demonstrated enrichment of 10 pathways previously reported in GBM studies [32–38], supporting the credibility of the selected gene set. Similarly, pathways enriched in GO and KEGG analyses, such as modulation of chemical synaptic transmission, the MAPK-signaling pathway, and the cMAP signaling pathway, have also been documented in GBM-related studie [33, 38, 39]. In the enrichment analysis of GO, synaptic membrane has been implicated in estrogen receptor beta (ERβ)-mediated signaling in glioma [40]. In contrast, monoatomic ion channel activity has not been reported in GBM and remains understudied in human cancers. Immune-related pathways were also analyzed. The pathogenesis of GBM was found to be closely linked to immune responses, particularly those involved in allograft rejection [37]. Allograft rejection may also function as a stratification biomarker in predicting the prognosis and immunotherapy efficacy [41]. The complement and coagulation cascades influence immune cell infiltration via AEBP1 regulation, potentially improving patient outcomes [33]. APG101, as reported by Gieffers, Christian et al., can alleviate CD95-mediated excessive apoptosis (such as graft versus host disease), offering therapeutic benefits in GBM [42]. Hu, Baoli et al. discovered that guadecitabine was shown to enhance antigen processing and presentation, promoting a more immunogenic phenotype in GBM cells [43]. These results emphasize the prognostic significance of immune pathways in GBM.

Among mRNAs related to hsa-miR-144-3p, five genes, namely, ARHGAP32, CALCRL, CD44, COTL1, and WNT5A—were significantly associated with prognosis (*p* < 0.01). Protein expression of COTL1 in the HPA did not align with current findings. However, other studies have shown high COTL1 expression in GBM, correlating with immune cell infiltration, immunomodulatory genes, and immune checkpoint markers, and associated with shorter survival in patients with glioma, GBM, and renal carcinoma [44]. No corresponding protein expression data for WNT5A were found in the HPA, but previous studies support its role in promoting invasive growth and recurrence by mediating glioma stem cell (GSC) differentiation [45]. These observations indicate that the protein expression patterns of COTL1 and WNT5A remain to be clarified. ARHGAP32 has not yet been reported in glioma studies, indicating a potential target for future research. CALCRL expression is elevated in LGG [46] but downregulated in GBM [47], suggesting a potential role in malignant progression. High expression of CD44 has been shown to enhance tumorigenicity in GBM, as demonstrated in mouse models by Anido, J. et al. [48]. Among the remaining 12 genes, CXCL16 promotes anti-apoptotic effects and cell proliferation in GBM [49]. DDIT4 may inhibit mTORC1 activity, and interfere with TMZ treatment, radiotherapy, or hypoxia-induced cell death [50]. FOSB has been associated with poor prognosis [51]. HOXA10 serves as a potential biomarker and therapeutic target in TMZ-resistant GBM [52]. RAB31 is upregulated in tumor tissues and identified as a key gene in GBM progression [53]. STC2 enhances GBM cell growth and invasiveness via increased expression and secretion [54]. TEAD1 modulates GBM cell migration in vitro [55] TIMP3 suppresses GBM-induced angiogenesis through upregulation [56]. TXNIP may contribute to treatment resistance following suppression of HOXA11 [57]. WLS plays a distinct role in GBM progression [58]. EPHA5 activation does not significantly influence tumor cell proliferation [59]. No reports have described the role of FJX1 in GBM; however, GEPIA database results reveal significant upregulation of FJX1 in tumor tissues, suggesting a potential role in tumorigenesis. Similar expression trends were observed for COTL1 and WNT5A. Collectively, these 17 genes are involved in GBM development, therapeutic response, and prognosis. The evidence highlights the therapeutic potential of hsa-miR-144-3p as a target in GBM, although its role in immune cell infiltration remains to be fully elucidated.

To comprehensively elucidate the functional role of hsa-miR-144-3p and its regulatory mechanisms on immune cells, KEGG and GO enrichment analyses were performed on 30 associated mRNAs. The results revealed significant enrichment in immune-related pathways, including complement and coagulation cascades, Fc gamma R-mediated phagocytosis, B cell receptor signaling pathway, leukocyte-mediated immunity, macrophage activation, myeloid leukocyte activation, immune receptor activity, and Toll-like receptor binding. These pathways have been well-documented to play critical roles in GBM immune modulation, immune evasion, immunotherapeutic response, and resistance mechanisms [33, 60–63]. This suggests that hsa-miR-144-3p may influence the regulation of local immune cell infiltration and potentially impact GBM prognosis.

Stromal score, immune score, and tumor purity were subsequently calculated to assess immune cell infiltration across subgroups stratified by PC1 levels. Significant differences in immune infiltration were observed between low- and high-PC1 groups, and stratification based on hsa-miR-144-3p–regulated mRNAs demonstrated predictive value for patient prognosis. Notably, a significant difference in PD-1 expression on intratumoral T cells was identified, while PDL1 expression on tumor cells showed no significant difference. This result suggests that hsa-miR-144-3p may regulate local immune activity by influencing T-cell populations, and that the effect of PD-1 expression on activated T cells may differ depending on the level of PD-L1 across the subgroups.

Considering the expression of PD-1 on the surface of T cells, a comprehensive immune infiltration analysis was performed using ssGSEA. Among the 22 immune cell types that were upregulated in the PC1-low group, several T-cell subsets were included, such as Activated CD8 + T cells, Activated dendritic cells, γδ T cells, T follicular helper cells, Activated CD4 + T cells, Effector memory CD4 + T cells, Th1 and Th17 cells, Natural killer T cells, Regulatory T cells, and Effector memory CD8 + T cells. These results highlight the clinical relevance of anti-PD-1 immunotherapy and identify immune cell subtypes potentially influenced by hsa-miR-144-3p in the context of GBM prognosis.

The elevated PD-1 expression, observed in prognostically favorable low PC1 group, may represent a compensatory response to immune suppression within the GBM tumor microenvironment [64]. It should be noted that the immune infiltration patterns observed in this study were inferred from bulk transcriptomic data using computational methods, including ssGSEA and CIBERSORT, rather than directly measured by experimental approaches. Therefore, these results should be interpreted with caution. Although such algorithms provide a useful framework for estimating immune-cell composition, their performance may be affected by transcriptomic complexity, overlapping gene signatures among immune and non-immune cell populations, and the intrinsic heterogeneity of GBM tissues. In particular, the highly heterogeneous cellular architecture of GBM, together with extracellular matrix and blood–brain barrier-related constraints on immune-cell trafficking, may limit the precision of computational immune deconvolution [65–67].

Nevertheless, the inferred immune patterns in our study are broadly consistent with known immune characteristics of GBM. GBM is widely recognized as an immunosuppressive tumor, characterized by restricted lymphocyte infiltration, abundant myeloid-cell populations, and profound functional exhaustion of tumor-infiltrating immune cells [64, 67, 68]. At the same time, previous studies have shown that although the blood–brain barrier restricts the trafficking of naïve T cells, activated T cells are relatively more capable of entering the central nervous system and tumor region [69]. This interpretation is consistent with the established view that GBM exhibits a predominantly immunosuppressive microenvironment, yet may still contain activated immune subsets in selected transcriptional states. In this context, the increased abundance of activated CD8 + T cells and macrophage-related signatures in the PC1-low group may reflect a relatively more immune-engaged transcriptional state rather than direct quantification of absolute immune-cell numbers. GBM still retains a certain potential for immune response, suggesting that it may benefit from immunotherapy. Our findings should be regarded as computationally inferred immune associations that provide biological hypotheses for further validation by immunohistochemistry, flow cytometry, or single-cell transcriptomic analyses.

We acknowledge that urothelial carcinoma differs substantially from GBM in terms of tumor microenvironment composition, mutational burden, and immune checkpoint dynamics [70, 71]. GBM is characterized by a highly immunosuppressive niche and limited T-cell trafficking, which may restrict the generalizability of findings derived from other tumor types [27, 64, 69]. Therefore, the IMvigor210 analysis should be interpreted as hypothesis-generating evidence rather than direct validation in GBM.

Given the association of hsa-miR-144-3p with immune-cell infiltration, PD-1 expression, and metabolic pathways linked to disulfidptosis, our findings may have meaningful implications for clinical and immunotherapeutic strategies in GBM. From a translational perspective, the PC1-based stratification model may have potential clinical utility in several aspects. First, it may serve as a transcriptomic tool for risk stratification by distinguishing patient subgroups with different survival outcomes. Second, because the PC1-low group displayed a relatively more immune-enriched transcriptional phenotype, including higher inferred immune scores and increased expression of selected immune checkpoint molecules, this model may help identify tumor states with greater immune involvement. Although this does not establish immunotherapy responsiveness, it may provide a framework for prioritizing patients for further immune profiling or biomarker-guided clinical investigation. More broadly, the miR-144-3p–related signature may contribute to future multimodal prognostic models integrating transcriptomic, immune, and metabolic features in GBM. The enrichment of activated T-cell subsets in the low-PC1 group, together with higher PD-1 expression, suggests that patients exhibiting this transcriptional phenotype may be more responsive to PD-1 blockade, as activated intratumoral T cells can retain therapeutic sensitivity despite PD-1 upregulation. Moreover, the metabolic alterations associated with disulfidptosis raise the possibility of combined metabolic–immune co-targeting approaches—for example, interventions modulating glucose metabolism in conjunction with immune checkpoint inhibition—which may enhance antitumor immunity in GBM. Therefore, the IMvigor210 analysis should be interpreted strictly as an exploratory cross-cancer assessment of immune association rather than as disease-specific clinical validation in GBM. Because urothelial carcinoma and GBM differ substantially in immune contexture, mutational landscape, and checkpoint biology, the predictive value observed in the IMvigor210 cohort cannot be directly extrapolated to GBM patients. Instead, this analysis provides only preliminary evidence that the has-miR-144-3p related transcriptional signature may capture immune-relevant biological features across tumor types. Future validation in GBM-specific immunotherapy cohorts will be necessary to determine whether this signature has true predictive value for treatment response in GBM.

Increasing evidence indicates that hsa-miR-144-3p may exert a dual biological role in GBM. Several studies have reported its tumor-suppressive function, showing that hsa-miR-144-3p inhibits GBM cell proliferation and invasion [72, 73]. In addition, findings from other cancer and inflammatory models suggest that hsa-miR-144-3p can modulate immune-related pathways, including cytokine signaling and T-cell activity [74, 75]. Together with our observation of its association with immune-cell infiltration and PD-1 expression, these data suggest that hsa-miR-144-3p may act not only as a tumor suppressor but also as an immune-modulatory miRNA in the GBM microenvironment. Furthermore, glucose metabolism is critically involved in immune cell activation [76].

To provide a balanced interpretation of our findings, several limitations of this study should be acknowledged. First, the sample size of the included cohorts is relatively limited, which may influence the robustness and generalizability of the findings. Second, although our analyses identified key disulfidptosis-related genes and microRNAs (miRNAs) associated with GBM, we did not perform in vitro or in vivo experimental validation to confirm their biological functions. In addition, immune cell infiltration was estimated using computational deconvolution methods based on bulk transcriptomic data rather than direct experimental measurement. Given the pronounced cellular heterogeneity of GBM and the potential influence of the blood–brain barrier on immune-cell trafficking, these inferred immune profiles should be interpreted cautiously. In addition, because this study integrated RNA-seq and microarray datasets generated from different platforms, residual platform-specific bias may still exist despite independent within-cohort analyses and intersection-based screening strategies.

Future studies will focus on validating the stability of hsa-miR-144-3p and its regulatory network through both in vitro and in vivo experiments, as well as in larger, independently annotated cohorts. Additional clinical variables will be incorporated to enable multivariate survival modeling, and single-cell transcriptomic data will be used to determine the cellular origins of its immune–metabolic regulatory activity. Furthermore, constructing and validating a multi-gene prognostic model centered on hsa-miR-144-3p, together with further mechanistic investigations, will help advance its translational potential in GBM.

Our integrative analysis suggests that hsa-miR-144-3p may participate in disulfidptosis regulation through coordinated modulation of cystine transport, NADPH metabolism, and mitochondrial redox homeostasis. Disulfidptosis is triggered by excessive cystine uptake mediated by the SLC7A11/SLC3A2 transporter system under NADPH-limited conditions, leading to intracellular disulfide stress and cytoskeletal collapse. In the present study, multiple hsa-miR-144-3p–associated genes, including ARHGAP32, TEAD1, CD44, ITGB8, and EGFR, showed positive correlations with SLC7A11 or SLC3A2, suggesting that hsa-miR-144-3p may influence system xCT activity and thereby modulate susceptibility to disulfidptosis. In addition, several negatively prognostic genes highly expressed in GBM (CD44, STC2, CXCL16, and FJX1) were positively correlated with glycolytic and pentose phosphate pathway enzymes, including GLUT1, GLUT3, G6PD, and GYS1, indicating enhanced NADPH-generating metabolic activity, which may buffer disulfide stress and suppress disulfidptosis. Conversely, multiple hsa-miR-144-3p associated genes showed negative correlations with mitochondrial complex I components such as NDUFA11, NDUFS1, and OXSM, suggesting altered mitochondrial redox metabolism and increased dependence on cytosolic NADPH production. Notably, many of these genes were differentially expressed in GBM and associated with prognosis, with CD44, STC2, CXCL16, and TEAD1 upregulated in tumors and linked to poor survival, whereas ARHGAP32 and EPHA5 were enriched in normal tissue and associated with favorable outcomes. Because metabolic reprogramming mediated by SLC7A11-dependent redox regulation and NADPH metabolism can influence oxidative stress, cytokine production, and immune cell recruitment, these findings suggest that hsa-miR-144-3p may contribute to immune modulation in glioblastoma through coordinated regulation of disulfidptosis-related metabolic pathways [77–79]. Although these results are based on correlative transcriptomic analyses, they provide a biologically plausible framework linking hsa-miR-144-3p to SLC7A11-dependent redox regulation, NADPH metabolism, and immune microenvironment remodeling.

In summary, our findings suggest that hsa-miR-144-3p is associated with immune-related transcriptional features in GBM and may represent a potential biomarker candidate for prognostic stratification. While exploratory cross-cancer analysis indicated limited immune-associated predictive signal, the clinical relevance of this signature for immunotherapy response in GBM remains uncertain and requires validation in disease-specific cohorts and experimental models.

## Conclusion

In summary, our findings suggest that hsa-miR-144-3p is associated with immune-related transcriptional features in GBM and may represent a potential biomarker candidate. While exploratory cross-cancer analysis indicated limited association with immunotherapy response, further validation in GBM-specific immunotherapy cohorts and experimental models is required.

## Electronic Supplementary Material

Below is the link to the electronic supplementary material.

Supplementary Material 1. Figure S1. PCA plots of TCGA and GSE4290. Figure S2. Selection of soft-thresholding power for WGCNA. Figure S3. Figures S3A-C PPI networks of cluster1, cluster2, and cluster3. Figure S4. Figures S4A-C KEGG enrichment analysis of Cluster 1, Cluster 2, and Cluster 3. Figure S5. Survival curves of 13 mRNAs. Figure S6. A. KEGG metabolic pathway enrichment analysis on the 30 mRNAs associated with hsa-miR-144-3p; B. Bar plot of immune cell composition. Figure S7. A. miR-144-3p signature scores in responders and non-responders; B. ROC curve for predicting immunotherapy response; C. Kaplan–Meier survival analysis of high vs. low score groups; D. Multivariate Cox analysis showing the signature score as an independent prognostic factor. Figure S8. A. Boxplots showing the expression of immune checkpoint–related genes across PC1 subgroups; B. Bar plot of immune cell composition; C. Boxplots illustrating differences in immune cell proportions between PC1 groups. Figure S9. A. Correlation between PC1 and immune score in the TCGA cohort; B. Correlation between PC1 and activated CD8⁺ T cell infiltration; C. Correlation heatmap of immune cell subsets

Supplementary Material 2. Table S1. DEGs in the TCGA dataset. Table S2. DEGs in the GSE4290 dataset. Table S3. DEGs in the GSE184472 dataset. Table S4. Intersection of DEGs in the TCGA dataset and the GSE4290 dataset. Table S5. miRNA and its related mRNA. Table S6. Diagnostic performance of candidate miRNAs. Table S7. Summary of survival analysis results. Table S8. Enrichment analysis of 28 immune cell types.

## Data Availability

The datasets generated and/or analysed during the current study are available in TCGA (https://portal.gdc.cancer.gov/), GEO (https://www.ncbi.nlm.nih.gov/geo), CGGA (https://www.cgga.org.cn) and IMvigor210 (https://github.com/SiYangming/IMvigor210CoreBiologies).
