# Supplementary figures and images for: Identification of hsa-miR-144-3p as a novel immunotherapeutic target for glioblastoma based on disulfidptosis-related analysis
Source: Discov Oncol. 2026 Apr 26;17:878. doi: 10.1007/s12672-026-05048-3 (PMC13247000; doi:10.1007/s12672-026-05048-3)

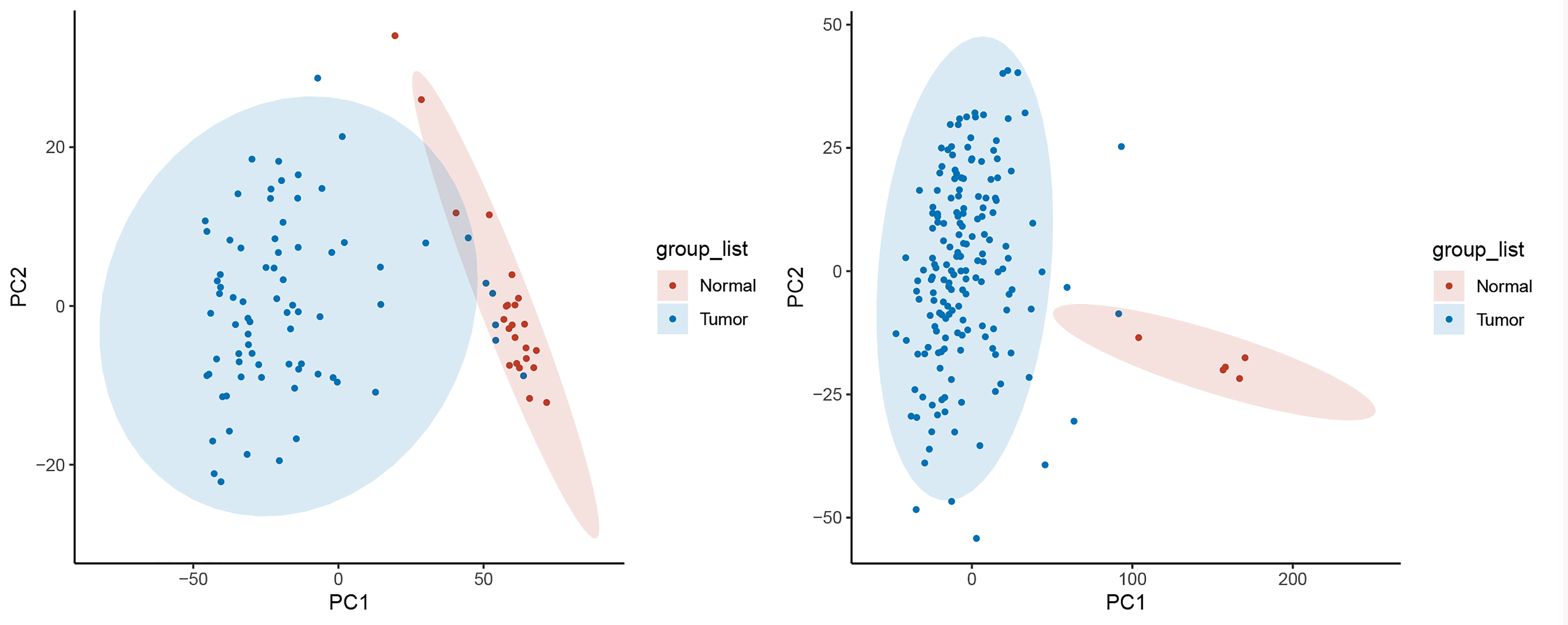

Supplement: Supplementary file 1 — Supplementary Material 1. Figure S1. PCA plots of TCGA and GSE4290. Figure S2. Selection of soft-thresholding power for WGCNA. Figure S3. Figures S3A-C PPI networks of cluster1, cluster2, and cluster3. Figure S4. Figures S4A-C KEGG enrichment analysis of Cluster 1, Cluster 2, and Cluster 3. Figure S5. Survival curves of 13 mRNAs. Figure S6. A. KEGG metabolic pathway enrichment analysis on the 30 mRNAs associated with hsa-miR-144-3p; B. Bar plot of immune cell composition. Figure S7. A. miR-144-3p signature scores in responders and non-responders; B. ROC curve for predicting immunotherapy response; C. Kaplan–Meier survival analysis of high vs. low score groups; D. Multivariate Cox analysis showing the signature score as an independent prognostic factor. Figure S8. A. Boxplots showing the expression of immune checkpoint–related genes across PC1 subgroups; B. Bar plot of immune cell composition; C. Boxplots illustrating differences in immune cell proportions between PC1 groups. Figure S9. A. Correlation between PC1 and immune score in the TCGA cohort; B. Correlation between PC1 and activated CD8⁺ T cell infiltration; C. Correlation heatmap of immune cell subsets [file 12672_2026_5048_MOESM1_ESM.zip › Supplementary Figures/Figure S1-R1.tif]

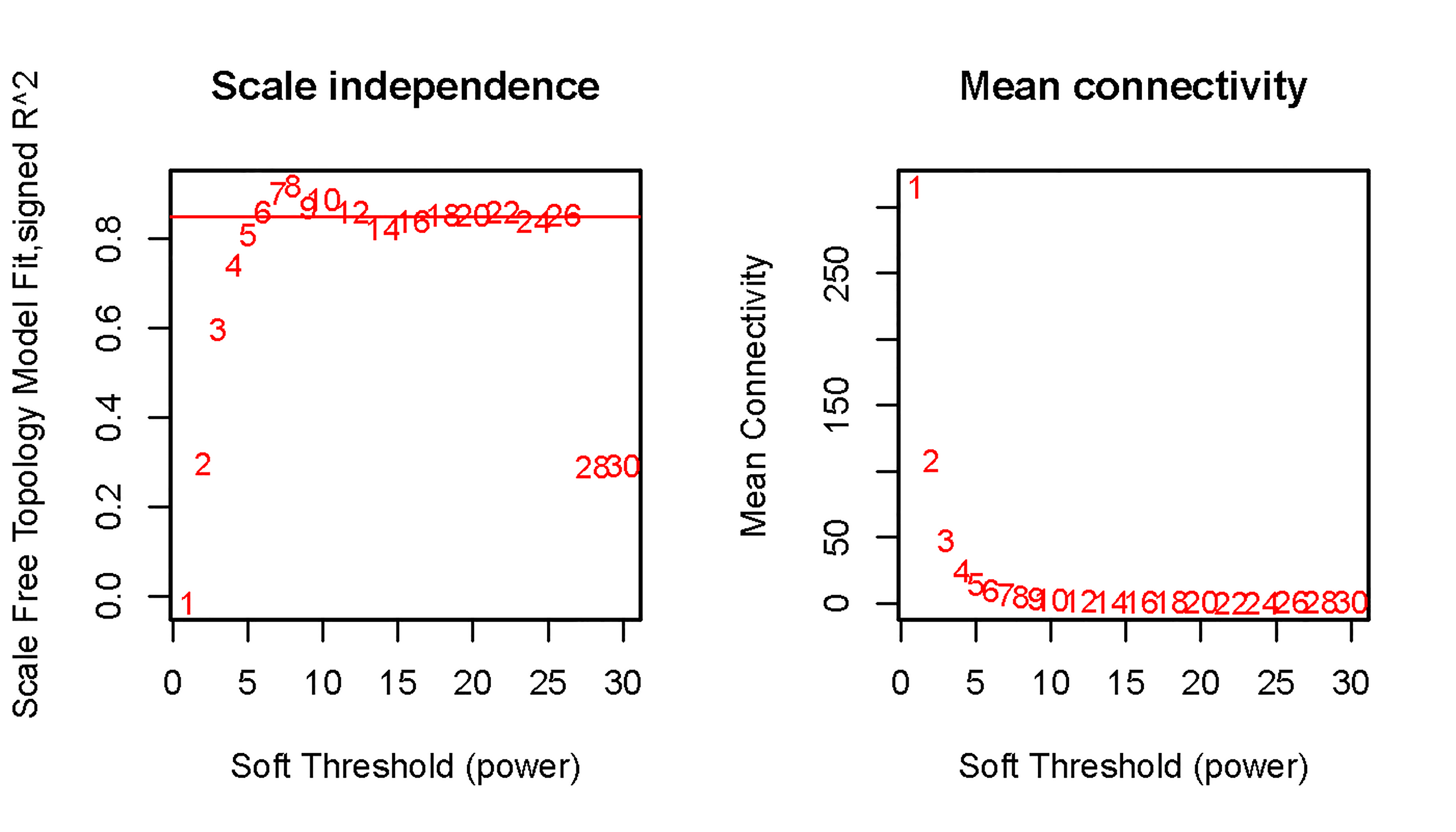

Supplement: Supplementary file 1 — Supplementary Material 1. Figure S1. PCA plots of TCGA and GSE4290. Figure S2. Selection of soft-thresholding power for WGCNA. Figure S3. Figures S3A-C PPI networks of cluster1, cluster2, and cluster3. Figure S4. Figures S4A-C KEGG enrichment analysis of Cluster 1, Cluster 2, and Cluster 3. Figure S5. Survival curves of 13 mRNAs. Figure S6. A. KEGG metabolic pathway enrichment analysis on the 30 mRNAs associated with hsa-miR-144-3p; B. Bar plot of immune cell composition. Figure S7. A. miR-144-3p signature scores in responders and non-responders; B. ROC curve for predicting immunotherapy response; C. Kaplan–Meier survival analysis of high vs. low score groups; D. Multivariate Cox analysis showing the signature score as an independent prognostic factor. Figure S8. A. Boxplots showing the expression of immune checkpoint–related genes across PC1 subgroups; B. Bar plot of immune cell composition; C. Boxplots illustrating differences in immune cell proportions between PC1 groups. Figure S9. A. Correlation between PC1 and immune score in the TCGA cohort; B. Correlation between PC1 and activated CD8⁺ T cell infiltration; C. Correlation heatmap of immune cell subsets [file 12672_2026_5048_MOESM1_ESM.zip › Supplementary Figures/Figure S2-R1.tif]

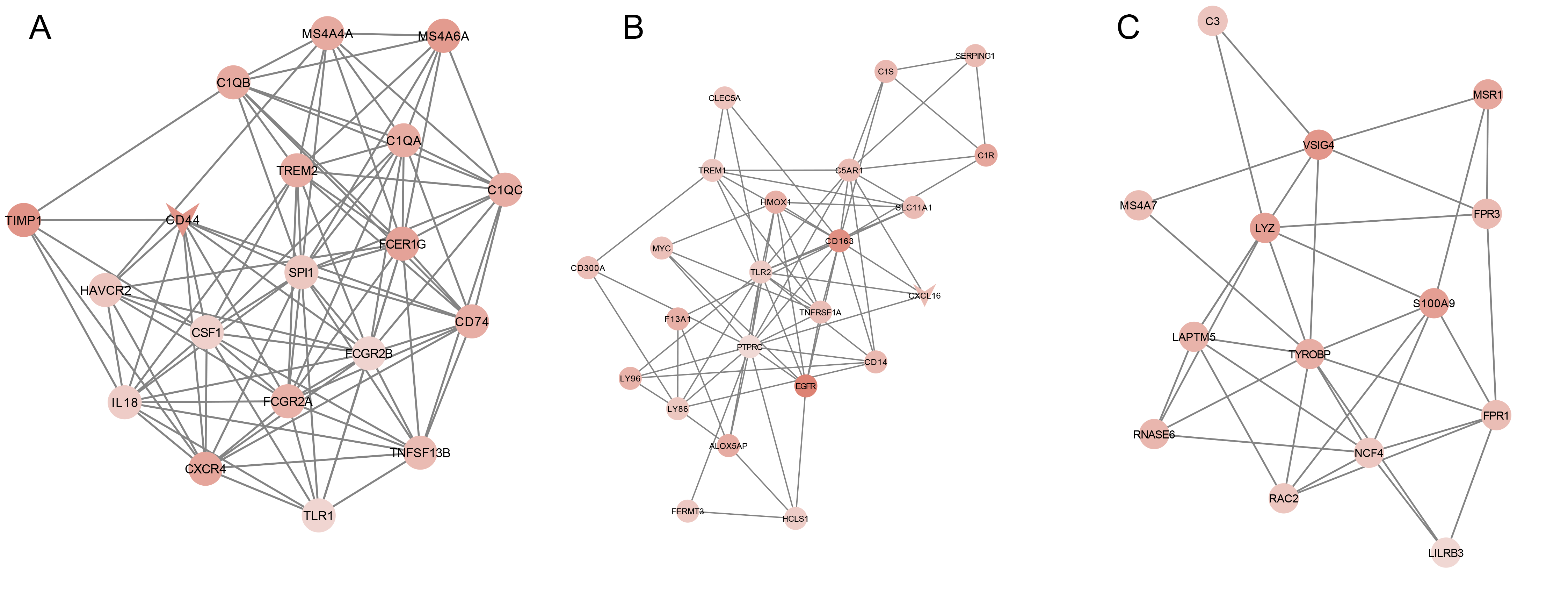

Supplement: Supplementary file 1 — Supplementary Material 1. Figure S1. PCA plots of TCGA and GSE4290. Figure S2. Selection of soft-thresholding power for WGCNA. Figure S3. Figures S3A-C PPI networks of cluster1, cluster2, and cluster3. Figure S4. Figures S4A-C KEGG enrichment analysis of Cluster 1, Cluster 2, and Cluster 3. Figure S5. Survival curves of 13 mRNAs. Figure S6. A. KEGG metabolic pathway enrichment analysis on the 30 mRNAs associated with hsa-miR-144-3p; B. Bar plot of immune cell composition. Figure S7. A. miR-144-3p signature scores in responders and non-responders; B. ROC curve for predicting immunotherapy response; C. Kaplan–Meier survival analysis of high vs. low score groups; D. Multivariate Cox analysis showing the signature score as an independent prognostic factor. Figure S8. A. Boxplots showing the expression of immune checkpoint–related genes across PC1 subgroups; B. Bar plot of immune cell composition; C. Boxplots illustrating differences in immune cell proportions between PC1 groups. Figure S9. A. Correlation between PC1 and immune score in the TCGA cohort; B. Correlation between PC1 and activated CD8⁺ T cell infiltration; C. Correlation heatmap of immune cell subsets [file 12672_2026_5048_MOESM1_ESM.zip › Supplementary Figures/Figure S3-R1.tif]

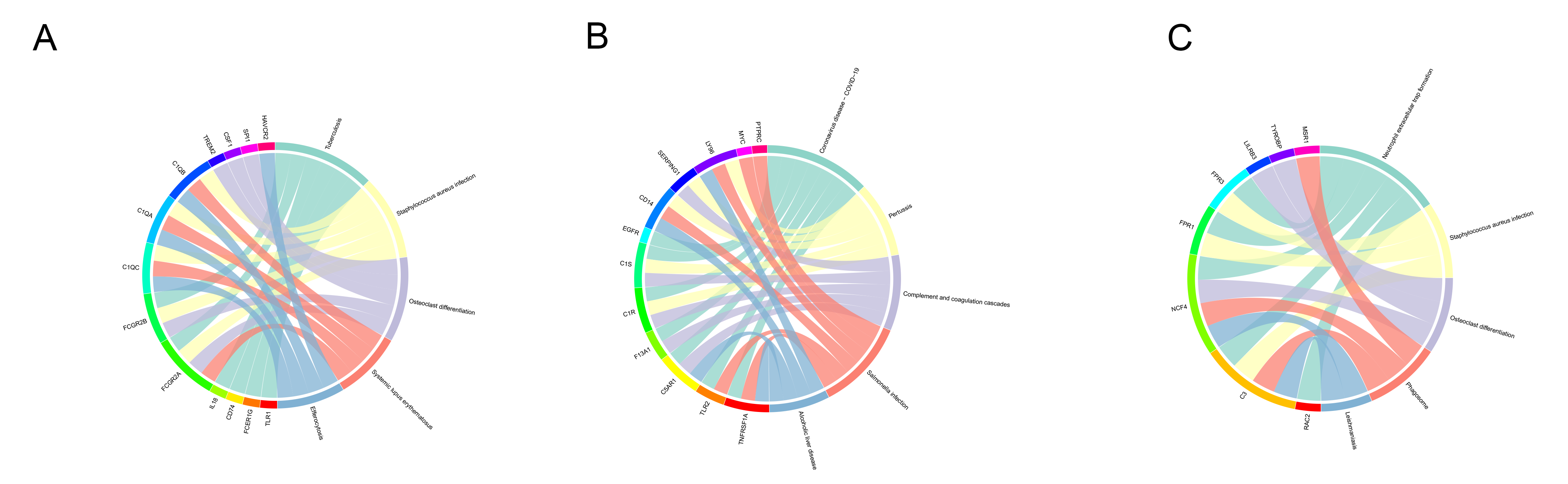

Supplement: Supplementary file 1 — Supplementary Material 1. Figure S1. PCA plots of TCGA and GSE4290. Figure S2. Selection of soft-thresholding power for WGCNA. Figure S3. Figures S3A-C PPI networks of cluster1, cluster2, and cluster3. Figure S4. Figures S4A-C KEGG enrichment analysis of Cluster 1, Cluster 2, and Cluster 3. Figure S5. Survival curves of 13 mRNAs. Figure S6. A. KEGG metabolic pathway enrichment analysis on the 30 mRNAs associated with hsa-miR-144-3p; B. Bar plot of immune cell composition. Figure S7. A. miR-144-3p signature scores in responders and non-responders; B. ROC curve for predicting immunotherapy response; C. Kaplan–Meier survival analysis of high vs. low score groups; D. Multivariate Cox analysis showing the signature score as an independent prognostic factor. Figure S8. A. Boxplots showing the expression of immune checkpoint–related genes across PC1 subgroups; B. Bar plot of immune cell composition; C. Boxplots illustrating differences in immune cell proportions between PC1 groups. Figure S9. A. Correlation between PC1 and immune score in the TCGA cohort; B. Correlation between PC1 and activated CD8⁺ T cell infiltration; C. Correlation heatmap of immune cell subsets [file 12672_2026_5048_MOESM1_ESM.zip › Supplementary Figures/Figure S4-R1.tif]

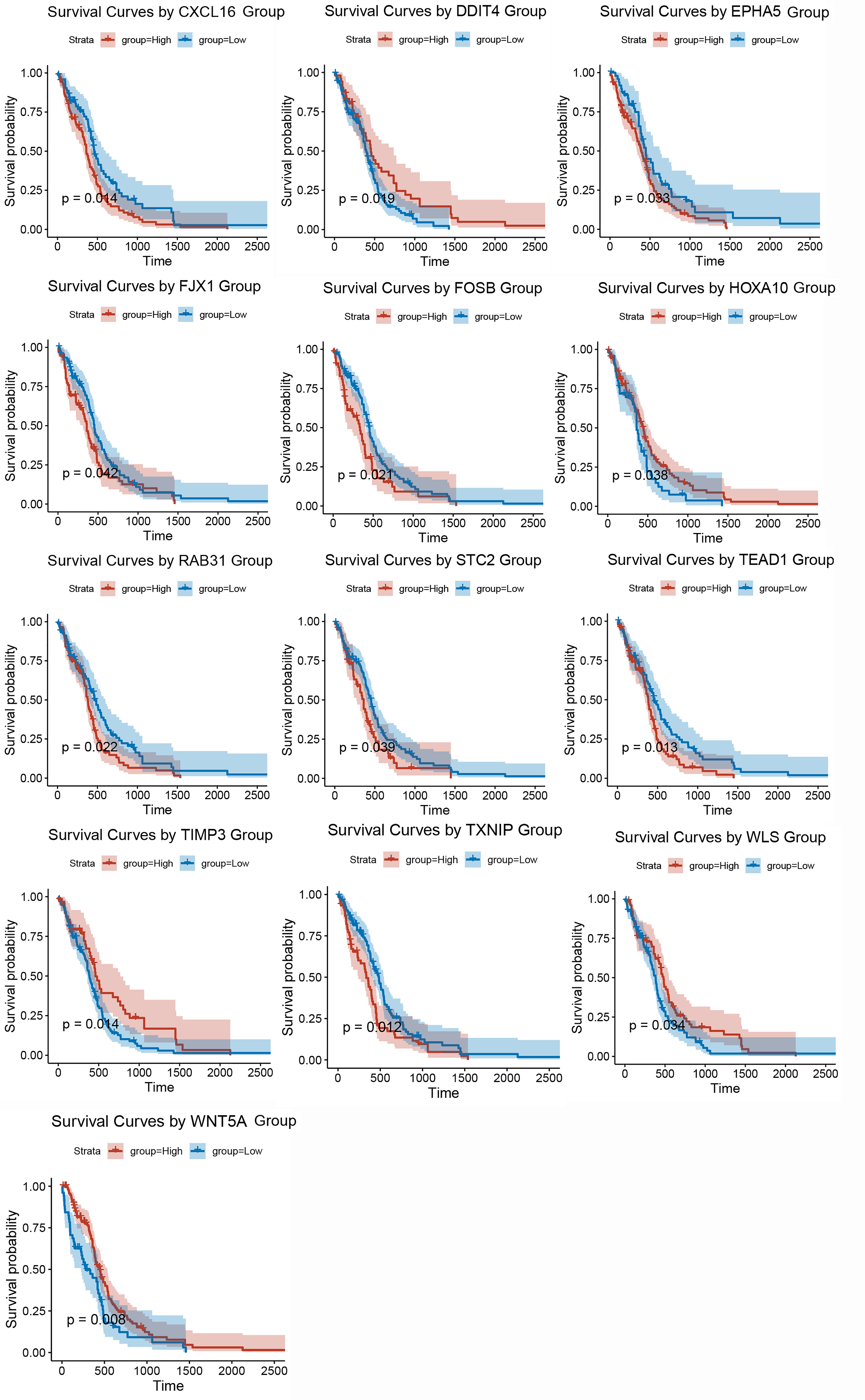

Supplement: Supplementary file 1 — Supplementary Material 1. Figure S1. PCA plots of TCGA and GSE4290. Figure S2. Selection of soft-thresholding power for WGCNA. Figure S3. Figures S3A-C PPI networks of cluster1, cluster2, and cluster3. Figure S4. Figures S4A-C KEGG enrichment analysis of Cluster 1, Cluster 2, and Cluster 3. Figure S5. Survival curves of 13 mRNAs. Figure S6. A. KEGG metabolic pathway enrichment analysis on the 30 mRNAs associated with hsa-miR-144-3p; B. Bar plot of immune cell composition. Figure S7. A. miR-144-3p signature scores in responders and non-responders; B. ROC curve for predicting immunotherapy response; C. Kaplan–Meier survival analysis of high vs. low score groups; D. Multivariate Cox analysis showing the signature score as an independent prognostic factor. Figure S8. A. Boxplots showing the expression of immune checkpoint–related genes across PC1 subgroups; B. Bar plot of immune cell composition; C. Boxplots illustrating differences in immune cell proportions between PC1 groups. Figure S9. A. Correlation between PC1 and immune score in the TCGA cohort; B. Correlation between PC1 and activated CD8⁺ T cell infiltration; C. Correlation heatmap of immune cell subsets [file 12672_2026_5048_MOESM1_ESM.zip › Supplementary Figures/Figure S5-R1.tif]

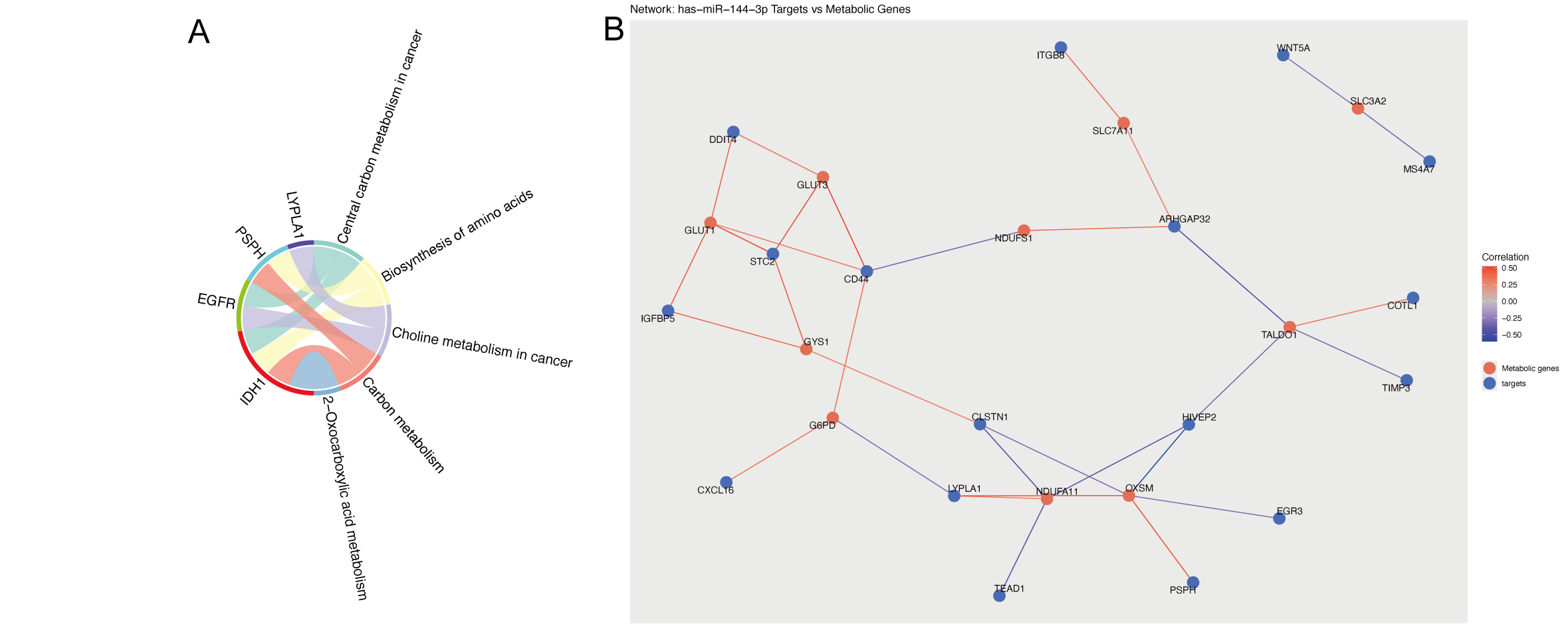

Supplement: Supplementary file 1 — Supplementary Material 1. Figure S1. PCA plots of TCGA and GSE4290. Figure S2. Selection of soft-thresholding power for WGCNA. Figure S3. Figures S3A-C PPI networks of cluster1, cluster2, and cluster3. Figure S4. Figures S4A-C KEGG enrichment analysis of Cluster 1, Cluster 2, and Cluster 3. Figure S5. Survival curves of 13 mRNAs. Figure S6. A. KEGG metabolic pathway enrichment analysis on the 30 mRNAs associated with hsa-miR-144-3p; B. Bar plot of immune cell composition. Figure S7. A. miR-144-3p signature scores in responders and non-responders; B. ROC curve for predicting immunotherapy response; C. Kaplan–Meier survival analysis of high vs. low score groups; D. Multivariate Cox analysis showing the signature score as an independent prognostic factor. Figure S8. A. Boxplots showing the expression of immune checkpoint–related genes across PC1 subgroups; B. Bar plot of immune cell composition; C. Boxplots illustrating differences in immune cell proportions between PC1 groups. Figure S9. A. Correlation between PC1 and immune score in the TCGA cohort; B. Correlation between PC1 and activated CD8⁺ T cell infiltration; C. Correlation heatmap of immune cell subsets [file 12672_2026_5048_MOESM1_ESM.zip › Supplementary Figures/Figure S6-R1.tif]

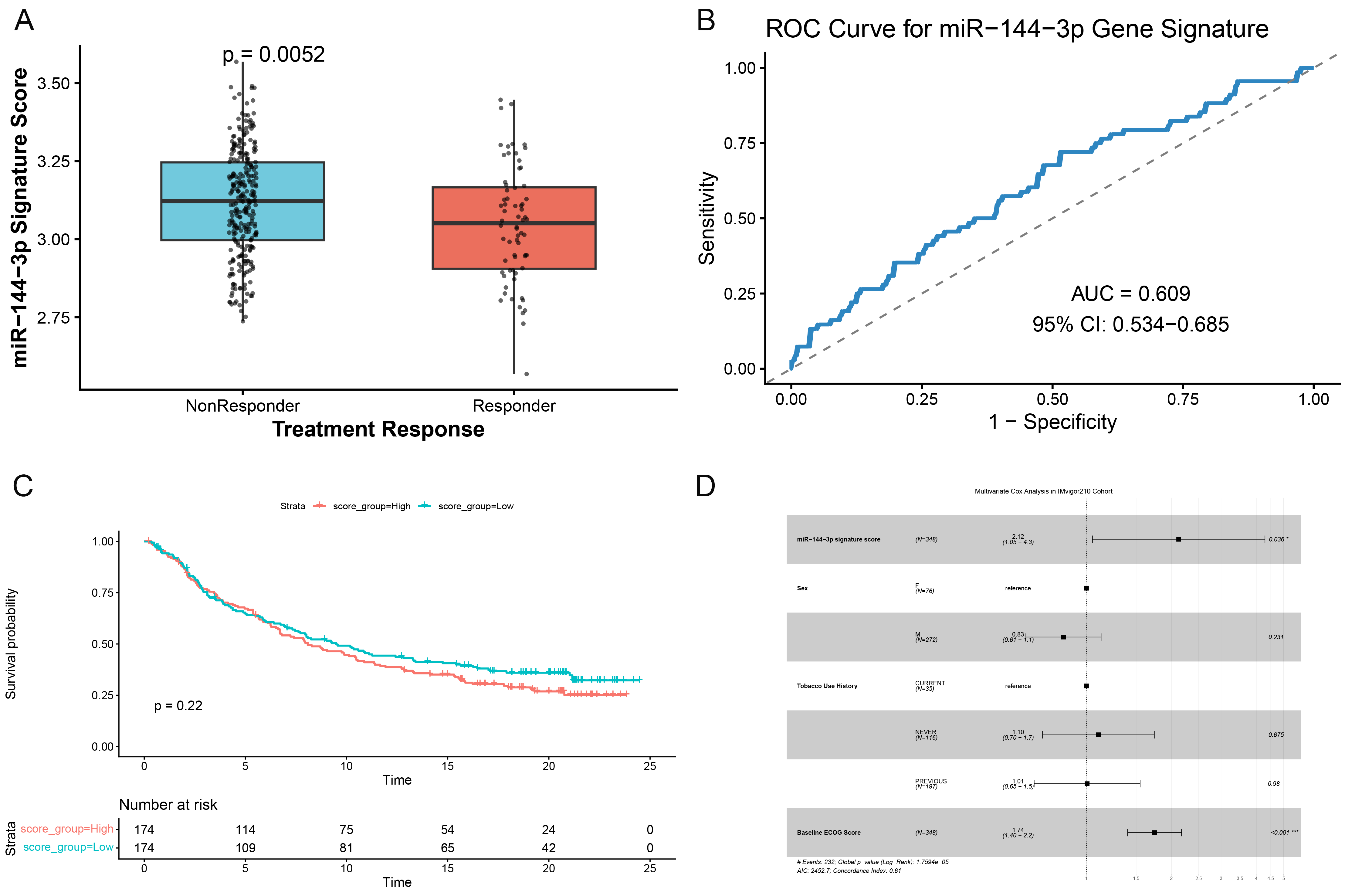

Supplement: Supplementary file 1 — Supplementary Material 1. Figure S1. PCA plots of TCGA and GSE4290. Figure S2. Selection of soft-thresholding power for WGCNA. Figure S3. Figures S3A-C PPI networks of cluster1, cluster2, and cluster3. Figure S4. Figures S4A-C KEGG enrichment analysis of Cluster 1, Cluster 2, and Cluster 3. Figure S5. Survival curves of 13 mRNAs. Figure S6. A. KEGG metabolic pathway enrichment analysis on the 30 mRNAs associated with hsa-miR-144-3p; B. Bar plot of immune cell composition. Figure S7. A. miR-144-3p signature scores in responders and non-responders; B. ROC curve for predicting immunotherapy response; C. Kaplan–Meier survival analysis of high vs. low score groups; D. Multivariate Cox analysis showing the signature score as an independent prognostic factor. Figure S8. A. Boxplots showing the expression of immune checkpoint–related genes across PC1 subgroups; B. Bar plot of immune cell composition; C. Boxplots illustrating differences in immune cell proportions between PC1 groups. Figure S9. A. Correlation between PC1 and immune score in the TCGA cohort; B. Correlation between PC1 and activated CD8⁺ T cell infiltration; C. Correlation heatmap of immune cell subsets [file 12672_2026_5048_MOESM1_ESM.zip › Supplementary Figures/Figure S7-R2.tif]

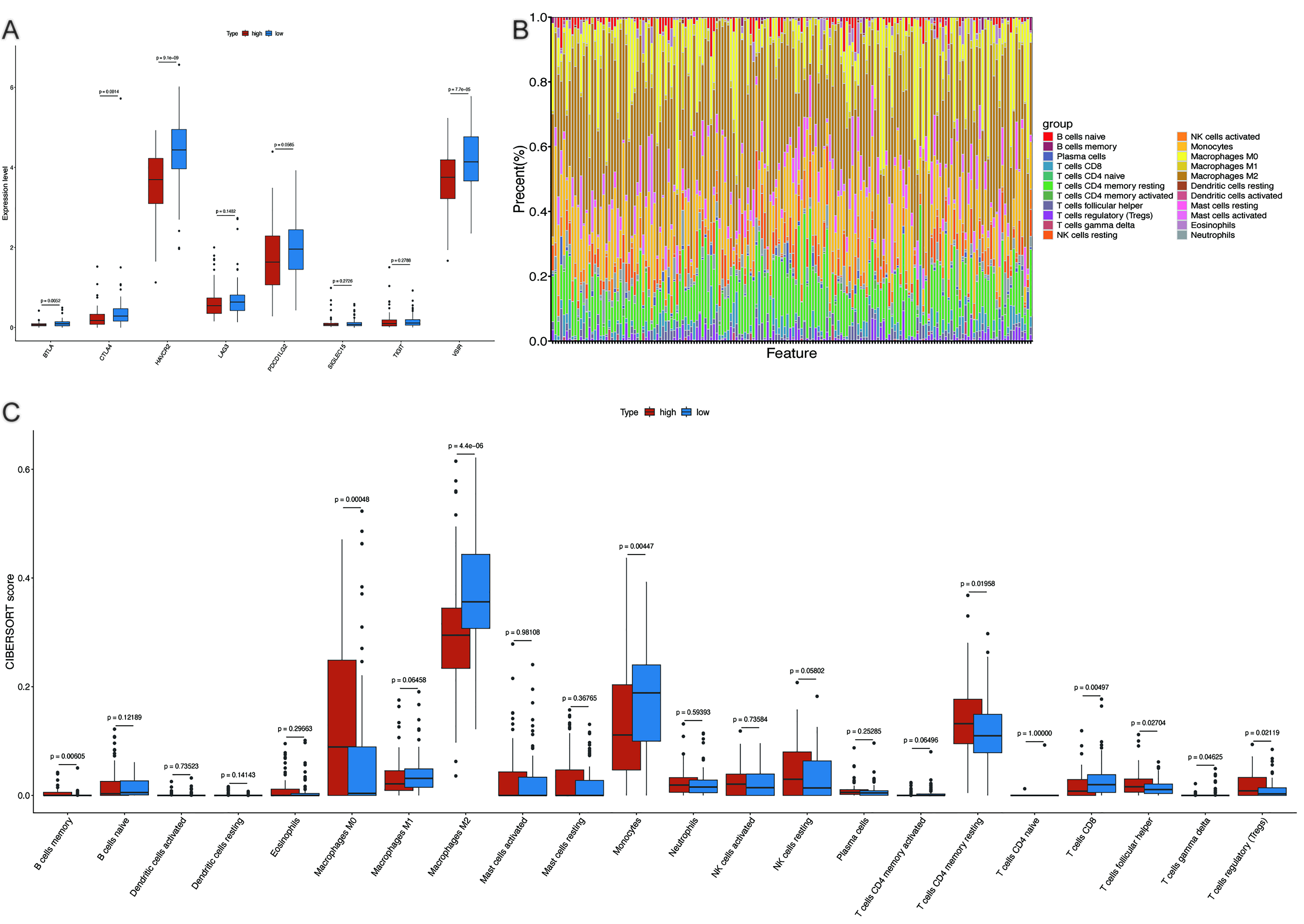

Supplement: Supplementary file 1 — Supplementary Material 1. Figure S1. PCA plots of TCGA and GSE4290. Figure S2. Selection of soft-thresholding power for WGCNA. Figure S3. Figures S3A-C PPI networks of cluster1, cluster2, and cluster3. Figure S4. Figures S4A-C KEGG enrichment analysis of Cluster 1, Cluster 2, and Cluster 3. Figure S5. Survival curves of 13 mRNAs. Figure S6. A. KEGG metabolic pathway enrichment analysis on the 30 mRNAs associated with hsa-miR-144-3p; B. Bar plot of immune cell composition. Figure S7. A. miR-144-3p signature scores in responders and non-responders; B. ROC curve for predicting immunotherapy response; C. Kaplan–Meier survival analysis of high vs. low score groups; D. Multivariate Cox analysis showing the signature score as an independent prognostic factor. Figure S8. A. Boxplots showing the expression of immune checkpoint–related genes across PC1 subgroups; B. Bar plot of immune cell composition; C. Boxplots illustrating differences in immune cell proportions between PC1 groups. Figure S9. A. Correlation between PC1 and immune score in the TCGA cohort; B. Correlation between PC1 and activated CD8⁺ T cell infiltration; C. Correlation heatmap of immune cell subsets [file 12672_2026_5048_MOESM1_ESM.zip › Supplementary Figures/Figure S8-R2.tif]

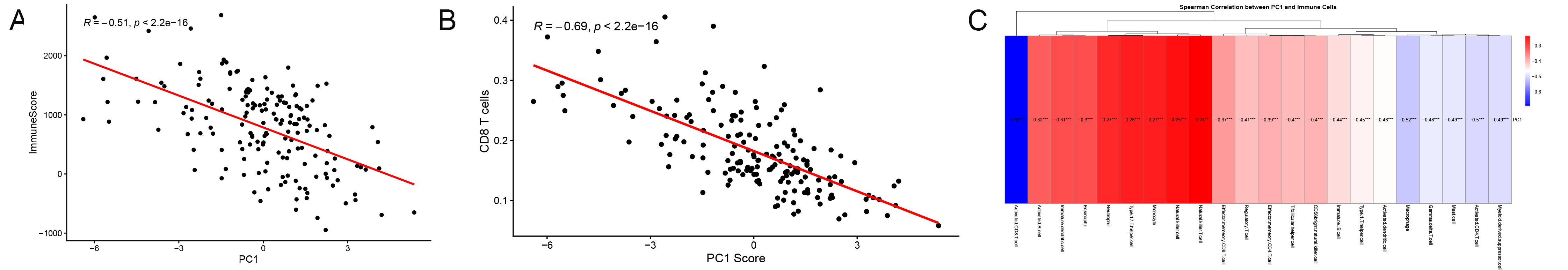

Supplement: Supplementary file 1 — Supplementary Material 1. Figure S1. PCA plots of TCGA and GSE4290. Figure S2. Selection of soft-thresholding power for WGCNA. Figure S3. Figures S3A-C PPI networks of cluster1, cluster2, and cluster3. Figure S4. Figures S4A-C KEGG enrichment analysis of Cluster 1, Cluster 2, and Cluster 3. Figure S5. Survival curves of 13 mRNAs. Figure S6. A. KEGG metabolic pathway enrichment analysis on the 30 mRNAs associated with hsa-miR-144-3p; B. Bar plot of immune cell composition. Figure S7. A. miR-144-3p signature scores in responders and non-responders; B. ROC curve for predicting immunotherapy response; C. Kaplan–Meier survival analysis of high vs. low score groups; D. Multivariate Cox analysis showing the signature score as an independent prognostic factor. Figure S8. A. Boxplots showing the expression of immune checkpoint–related genes across PC1 subgroups; B. Bar plot of immune cell composition; C. Boxplots illustrating differences in immune cell proportions between PC1 groups. Figure S9. A. Correlation between PC1 and immune score in the TCGA cohort; B. Correlation between PC1 and activated CD8⁺ T cell infiltration; C. Correlation heatmap of immune cell subsets [file 12672_2026_5048_MOESM1_ESM.zip › Supplementary Figures/Figure S9-R2.tif]
